# Comparative study between photodynamic therapy with urucum + Led and probiotics in halitosis reduction–protocol for a controlled clinical trial

**DOI:** 10.1371/journal.pone.0247096

**Published:** 2021-05-14

**Authors:** Pamella de Barros Motta, Lara Jansiski Motta, Ana Carolina Costa da Mota, Marcela Letícia Leal Gonçalves, Tamiris Silva, Marcos Momolli, David Casimiro de Andrade, Kristianne Porta Santos Fernandes, Raquel Agnelli Mesquita-Ferrari, Alessandro Melo Deana, Mark Wainwright, Renato Araujo Prates, Anna Carolina Ratto Tempestini Horliana, Sandra Kalil Bussadori

**Affiliations:** 1 Post Graduate Program in Biophotonics Applied to Health Sciences, Universidade Nove de Julho, São Paulo, SP, Brazil; 2 Dentistry College, Universidade Metropolitana de Santos (UNIMES), Santos, SP, Brazil; 3 School of Dentistry, University of Porto, Porto, Portugal; 4 School of Pharmacy and Biomolecular Sciences, Liverpool John Moores University, Liverpool, United Kingdom; State University of Ponta Grossa, BRAZIL

## Abstract

**Background:**

Halitosis is a term that defines any foul odor emanating from the oral cavity. The origin may be local or systemic. The aim of the proposed protocol is to determine whether treatment with antimicrobial photodynamic therapy (aPDT) and treatment with probiotics are effective at eliminating halitosis.

**Materials and methods:**

Eighty-eight patients, from 18 to 25 years old with a diagnosis of halitosis (H_2_S≥112 ppb, determined by gas chromatography) will be randomly allocated to four groups (n = 22) that will receive different treatments: Group 1 –treatment with teeth brushing, dental floss and tongue scraper; Group 2 –brushing, dental floss and aPDT; Group 3 –brushing, dental floss and probiotics; Group 4 –brushing, flossing, aPDT and probiotics. The results of the halimetry will be compared before, immediately after, seven days and thirty days after treatment. The microbiological analysis of the coated tongue will be performed at these same times. The normality of the data will be determined using the Shapiro-Wilk test. Data with normal distribution will be analyzed using analysis of variance (ANOVA). Non-parametric data will be analyzed using the Kruskal-Wallis test. The Wilcoxon test will be used to analyze the results of each treatment at the different evaluation periods.

**Clinical trail registration:**

NCT03996044.

## Introduction

Halitosis is a term used to define any foul odor emanating from the oral cavity, the origin of which may be local or systemic [1]. This oral health problem is the third major reason for patients to seek treatment at a dental office [2]. Halitosis is related to the presence of volatile sulfur compounds (VSCs), such as hydrogen sulfide (H_2_S), methanethiol (CH_3_SH) and dimethyl sulfide (CH_3_SCH_3_) [3–6].

Different methods are used for the diagnosis of halitosis. The clinical evaluation (organoleptic test) is a subjective method, in which the breath exhaled from the mouth is smelled by a professional, followed by the quantification of the odor using a scale. Gas chromatography is the most appropriate method for the diagnosis of halitosis of any origin, as it enables the measurement and separation of the three main gases (hydrogen sulfide, resulting from the tongue coating; methanethiol, from periodontal pockets; and dimethyl sulfide, a gas with systemic origin) [7–9].

Although the etiology of halitosis is complex, anaerobic bacteria are considered to be the main cause [7]. The prevalence of halitosis is high, and rates higher than 50% have been found in the literature [10]. Conventional treatment consists of the use of toothpastes and mouthwashes containing bactericidal substances, the use of a tongue scraper, the treatment of dental caries and periodontal disease and the control of xerostomia [11]. Some studies suggest that amine fluoride has a positive effect on diminishing halitosis [12]. Alternative treatments, such as antimicrobial photodynamic therapy (aPDT) [13–15] and probiotics have also been employed in an attempt to control halitosis [10,11,16].

aPDT involves the use of a photosensitizing agent and the presence of light, which produces free oxygen radicals, leading to cell death. For halitosis, a condition for whose main etiological factor is anaerobic bacteria, aPDT has achieved positive results with the use of laser in the red portion of the spectrum and methylene blue as a photosensitizer, leading to a reduction in hydrogen sulfide and the bacterial load on the dorsum of the tongue [13,15,17].

Probiotics are microorganisms that are beneficial to health when absorbed by the host. These microorganisms have been used in food, fermented products and pharmaceutical formulations [18]. Studies have shown positive effects of the use of probiotics regarding the control of halitosis, suggesting that these microorganisms favor the elimination of undesirable microorganisms and promote the recolonization of the microbiota of the individual [16,19,20].

The advantages of these alternative approaches are a reduction in harm to tissues and the avoidance of bacterial resistance. Thus, these methods can be used to develop an effective, lasting treatment for halitosis by eliminating the anaerobic bacteria related to this condition and possibly recolonizing the microbiota on the dorsum of the tongue to improve the quality of life of affected individuals. Indeed, halitosis is considered an important social problem, as it can interfere with interpersonal relations and cause concerns regarding one’s physical health, which can lead to psychological problems and a social barrier [21].

The proposed project is justified due to the lack of studies that evaluate the reduction in halitosis using aPDT and probiotics. We propose using urucum as the photosensitizer and light-emmitting diode (LED) as the light source. This allows greater access to the treatment, seeing as most dentists already have LEDs in the office because of the photopolymerization of composite resins.

## Materials and methods

The protocol was based on results that were previously published in the literature [15,22–25]. A randomized, controlled, clinical trial will be conducted. After receiving clarifications regarding the objectives and procedures of the study, the volunteers will agree to participate by signing a statement of informed consent.

**Fig 1:**
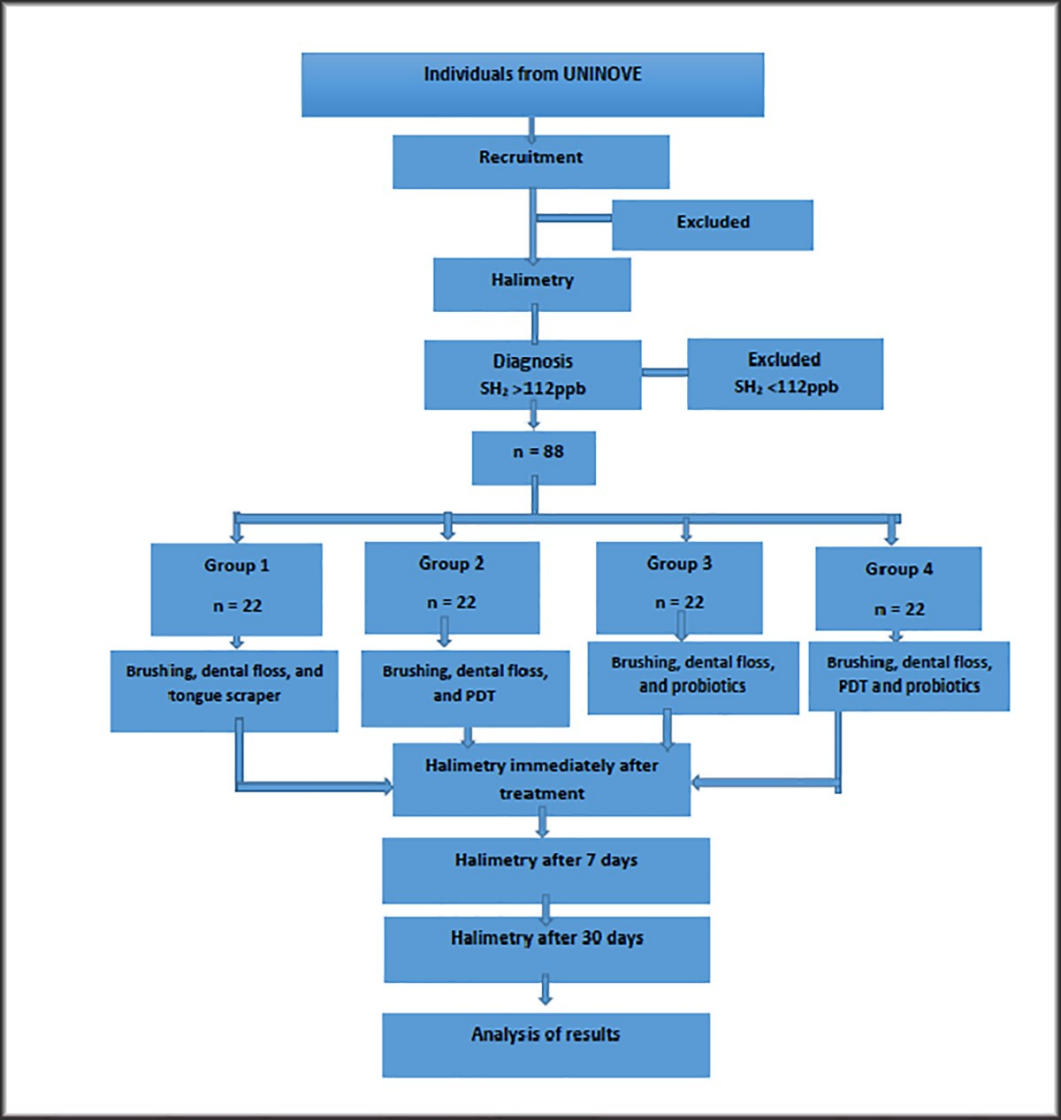
Flowchart of activities.

Fifty-two individuals with a diagnosis of halitosis (H_2_S) ≥ 112 ppb) will be selected. Halitosis will be diagnosed using gas chromatography with Oralchroma^TM^. This device has been chosen because it divides gases according to their origin, and we intend to select patients who have halitosis resulting from the tongue coating (high H_2_S). The participants will be divided in four groups (n = 22) (Fig 1). Patients will be randomized by block randomization into the groups, according to the treatment to be performed. To randomly distribute the subjects, a draw will be made with numbers. As the numbers are drawn, they will compose the experimental groups. Opaque envelopes will be identified with each number and inside it a sheet containing the information of the corresponding experimental group will be inserted according to the order obtained in the draw. The envelopes will be sealed and will remain sealed in numerical order in a safe place until the procedures are made. The person in charge of the drawing process will not be envolved in the other parts of the study. The groups are corresponding to the treatments, as follows: Group 1 –treatment with brushing, dental floss and a tongue scraper; Group 2 –brushing, dental floss and aPDT applied to the posterior and middle thirds of the tongue; Group 3 –brushing, dental floss and probiotics; Group 4 –brushing, flossing, aPDT and probiotics. The results of the halimetry will be compared before, immediately after, seven days and thirty days after treatment. The microbiological analysis of the tongue coating will be performed at these same times. The quantitative analysis will be conducted using real-time PCRq. The control periods of seven and thirty days are compatible to how dentists could apply and control the treatments clinically. The seven days’ control will be important to evaluate the effectiveness of the oral hygiene explanations and of the aPDT, showing if there will be a need to re-explain hygiene concepts or do another aPDT session after 7 days, making it a weekly clinical procedure in persistent cases of halitosis. The 30 days’ control will be important to evaluate the action of probiotics in the reduction of this conduction, as well as check whether the hygiene instructions are still being respected after this period.

### Inclusion criteria

Male and female individuals between 18 and 25 years of age with a diagnosis of halitosis using gas chromatography with OralChroma^TM^ of H_2_S ≥ 112 ppb. The consideration of this gas is due to the fact that only patients with halitosis resulting from bacteria of the tongue coating will be selected.

### Exclusion criteria

Individuals with dentofacial anomalies (hare lip, cleft lip/palate), those undergoing orthodontic or orthopedic treatment, those in treatment for cancer, those with carious lesions or periodontal diseases, those with systemic (gastrointestinal, renal or hepatic) disorders, those having taken antibiotics in the month prior to the study, pregnant women, individuals with fissured tongue and smokers will be excluded from the study.

### Ethics and dissemination

This study has been approved by the Ethics Committee of *Universidade Nove de Julho* (UNINOVE) under process number 3.669.442 and will be conducted in accordance with the norms governing research involving human subjects stipulated in Resolution 466/2012 of the Brazilian National Board of Health. Changes in the protocol will be reported to this same committee.

### Informed consent process

Individuals deemed eligible will receive clarifications regarding the objectives and procedures of the study and those who agree to participate will sign a statement of informed consent. The identity of all individuals will be preserved throughout all stages of the research. No harms are expected.

### Procedures

This protocol is in accordance with the 2013 Standard Protocol Items: Recommendations for Interventional Trials (SPIRIT) Statement and the SPIRIT checklist can be found as an additional file.

### Evaluation of coated tongue

Tongue coating will be quantified using the Coated Tongue Index (CTI) proposed by Shimizu et al. [26]. The tongue is divided into nine parts and a score is attributed to each part: 0 –absence of coated tongue; 1 –coated tongue with visible papillae; 2 –thickness of coating blocks the visualization of papillae. The scores will be summed, divided by 18 and multiplied by 100, giving a final index ranging from 0 to 100%.

### Halimetry

The collection of air from the oral cavity will be performed following the manufacturer’s instructions (Oral Chroma^TM^). Being a machine, this device is a blind assessor. The participant will be instructed to rinse his/her mouth with cysteine (10 mM) for one minute and then remain with his/her mouth closed for one minute. The syringe from the same manufacturer will be inserted into the mouth and the participant will remain with the mouth closed for one minute, breathing through the nose and without touching the tongue to the syringe. The plunger will be withdrawn; the air will be pushed back into the mouth and the plunger will be withdrawn a second time to fill the syringe with the breath sample. The tip of the syringe will be cleaned with gauze to remove the saliva and the injection needle will be placed on the syringe. The plunger will be adjusted to 0.5 ml and the sample will be injected in the entry port of Oral Chroma^TM^ in a single motion.

The following VSCs will be analyzed by the system:

1. Hydrogen sulfide–originating mainly from bacteria on the dorsum of the tongue (values higher than 112 ppb indicate halitosis);
2. Methanethiol–higher predominantly in periodontal pockets (values up to 26 ppb are considered normal). Periodontal disease typically results in a high methanethiol/hydrogen sulfide ratio [>3:1]);
3. Dimethyl sulfide–of either a periodontal or systemic (intestinal, hepatic, pulmonary) origin. This gas can also be temporarily caused by the ingestion of certain foods and beverages. It is possible to distinguish dimethyl sulfide of an oral or systemic origin through the comparison of the results of the halimeter testing with and without cysteine challenge (10 mM of cysteine = 16 mg of cysteine in 100 ml of distilled water [16 mg%]). The perception threshold of dimethyl sulfide is very low (8 ppb).

Other non-VSC odors may also appear at a peak prior to the theoretical first peak, which is that of hydrogen sulfide. To avoid confounding the device, participants will be given the following instructions: refrain from ingesting food with garlic, onion or strong spices; avoid the consumption of alcohol and the use of an antiseptic mouthwash in the 48 hours prior to the evaluation; refrain from drinking coffee, eating breath mints and chewing gum, as well as the use of personal and oral hygiene products (aftershave, deodorant, perfume, creams or tonic water) on the day of the evaluation; and refrain from eating anything in the two hours prior to the evaluation.

### Microbiological analysis

For the mirobiological analysis, the technique described by Ye *et al*. [27] will be used. Samples of the tongue coating will be collected from the participants before, immediately after and thirty days after treatment, in all groups. It will be scraped with back and forth movements with a tongue scraper and transferred to a 1 ml Tris-EDTA buffer. The samples will then be stored at a −80° C freezer. The microbiome and the variations between the communities will be analyzed using methods of pyrosquencing and metagenomics of the 16S rRNA gene. The E.Z.N.A.® soil DNA kit (Omega Bio-tek, Norcross, GA, USA) will be used to extract microbial DNA. A UV-vis spectrophotometer (NanoDrop 2000, Thermo Scientific, Wilmington, USA) will be used to determine the final DNA concentration and purification. DNA quality will be assessed by electrophoresis on a 1% agarose gel.

The hypervariable V3-V4 regions of the bacterium’s 16S rRNA gene will be amplified with primers 338F (5′-ACTCCTACGGGAGGCAGCAG-3 ′) and 806R (5 ′ GGACTACHVGGGTWTCTAAT-3 ′) using the thermocycler system, USA 9700 (GeneAmp, USA 9700). For PCR reactions, the following steps will be performed: three minutes of denaturation at 95° C, 27 cycles of 30 s to 95° C, 30 s of annealing at 55° C, 45 s of elongation at 72° C and an extension final at 72° C for 10 min. The PCR reactions will be performed in triplicate using a 20 μl aliquot of the mixture, containing 4 μl of 5 × FastPfu Buffer, 2 μl of 2.5 mM dNTPs, 0.8 μl of each primer (5 μM), 0.4 μl of FastPfu Polymerase, and 10 ng of template DNA. The AxyPrep DNA Gel Extraction Kit (Axygen Biosciences, Union City, CA, USA) will be used to extract and purify the PCR products resulting from the 2% agarose gel. Further quantification will be performed using QuantiFluor™-ST (Promega, USA). The purified amplicons will be grouped in equimolar quantities, and the paired sequencing (2 × 300) will be performed on the Illumina MiSeq platform (Illumina, San Diego, USA), according to the standard protocols provided by Majorbio Bio-Pharm Technology Co. Ltd. (Shanghai, China). Sequencing data processing Raw fastq files will be demultiplexed, filtered for quality by Trimmomatic and merged using the FLASH software. Operational taxonomic will be grouped with 97% similarity cut using UPARSE (version 7.1, http://drive5.com/uparse/), and chimeric sequences will be identified and removed using UCHIME (version 4.1). The taxonomy of each sequence of the 16S rRNA gene will be analyzed with the RDP Classifier algorithm (http://rdp.cme.msu.edu/) against the Silva (SSU123) and 16S rRNA databases. The data will then be analyzed using the free online platform Majorbio I-Sanger Cloud (www.i-sanger.com). Representative sequences will be assigned from the phylum to the species level. Shannon’s alpha (α) index will be used to assess the bacterial richness and diversity of the tongue between samples. The Venn diagrams will be implemented by the Venn Diagram (version 1.6.20). The beta diversity analysis will be performed using the weighted distance matrices UniFrac and Bray-Curtis to compare the results of the main coordination analysis (PCoA). The R-forge community ecology package (Vegan 2.0 package) will be used to generate the PCoA figures and heat map, and perform the redundancy analysis (RDA) with Monte Carlo permutations (permu = 999). The variance inflation factor (VIF) will be measured to assess the collinearity between the various environmental factors (VSC gas concentration values). Environmental factors with VIF> 10 will be removed after Spearman’s correlation analysis [27]. This same study [27] has also shown that genera *Prevotella*, *Alloprevotella*, *Leptotrichia*, *Peptostreptococcus and Stomatobaculum* exhibited significantly higher relative percentages in halitosis samples, when compared to healthy samples. As a result, we expect these will be the main types of bacteria to be found.

The purpose of the microbiological evaluation will be to determine the effectiveness of hygiene control, aPDT and probiotics for the treatment of halitosis. The researcher in charge of conducting the microbiological analysis will not be aware of which group the samples belong to.

### Antimicrobial photodynamic therapy

For aPDT, urucum will be used as photosensitizer (PS), associated with a blue LED, following an already published protocol [28]. The absorbance of urucum has been tested in a previous study by Gonçalves *et al*. [29] and it showed a peak of absorption close to the 460nm (≅400nm-510nm), which is one of the peaks of wavelength generated by the LED that will be used in this study. In addition to that, it has been shown that the urucum generates singlet oxygen when irradiated with a blue light in a dose-dependent manner, i.e., the higher the concentration the extract, the greater is the production of singlet oxygen, which is necessary for PDT.

**Fig 2:**
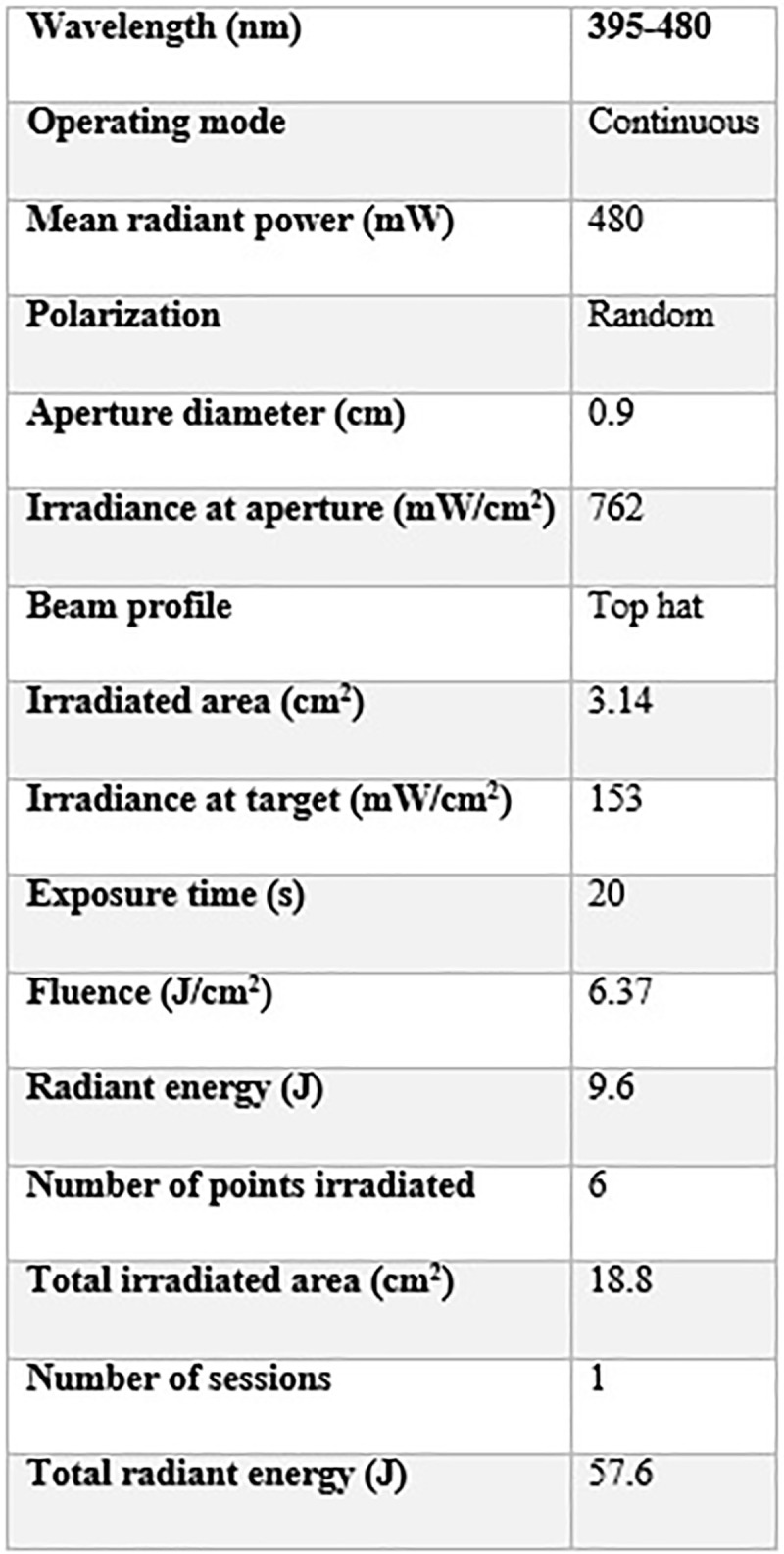
LED parameters.

aPDT will be performed with a LED device (Valo Cordless Ultradent^®^), which is a common dental office device, coupled to a radiometer and operating in the 395 to 480nm spectrum, with irradiance of 450mW/cm. Only the volunteer and operator will be present at the time of aPDT and both will be using protective eyewear. The active tip of the LED will be covered with plastic wrap (PVC) to avoid cross-contamination and for the purposes of hygiene. The operator will be duly appareled. One session of aPDT will be performed with the urucum at a concentration of 20% (Fórmula e Ação^®^) in spray form. The PS will be applied in sufficient quantity to cover the middle and posterior of the tongue (five sprays) for two minutes of incubation. The excess will be removed with an aspirator and the PS will maintain the surface moist, without the need for water. Six points will be irradiated, with a distance of 1 cm between points considering the spread of the light and effectiveness of aPDT. The device will be calibrated with wavelength between 395 and 480 nm, energy of 9.6 J and exposure time of 20 seconds per point. The light will be irradiated to form a 2 cm halo in diameter per point. Fig 2 displays the LED parameters to be used in the study.

### Tongue scraping

Tongue scraping will be performed by the same operator in all participants. Posteroanterior movements will be made with the scraper on the dorsum of the tongue, followed by the cleaning of the scraper with gauze. This procedure will be performed ten times on each participant to standardize the mechanical removal of tongue coating.

### Treatment with probiotics

Capsules compounded at a pharmacy containing the strain *Lactobacillus salivarius* WB21 (6.7 x 108 CFUs) and xylitol (280 mg) will be used. Forty-two capsules will be given to each participant, who will be instructed to ingest one capsule three times per day, after meals, for 14 days.

### Brushing with toothpaste containing amine fluoride

All 52 participants will be instructed during a lecture on how to brush with a toothpaste containing amine fluoride (Elmex^®^), and use dental floss three times per day, after meals, for 30 days. The Bass technique will be taught, with the bristles positioned at an approximately 45° angle to the gum line on both the free and proximal faces, performing short and slightly circular vibratory movements [30,31].

### Sample calculation

The sample size calculation was determined in regards to the primary outcome of the study: H_2_S and it was based on the data from Costa da Mota *et al*. (Effect of photodynamic therapy for the treatment of halitosis in adolescents—a controlled, microbiological, clinical trial) [15]. Our initial sample size estimation was of 15 subjects per group for a significant level of 0.05 and an estimated test power of 80%. To account for the possible non-parametric distribution of the data, 15% more subjects must be added to each group [32]. Another 25% will be added to account for possible dropouts, resulting in 22 subjects per group. G*Power 3.1.9.6 was used to perform the calculations.

### Organization and statistical treatment of data

The data from the Oral Chroma^TM^ device will be analyzed for normality using the Shapiro-Wilk test. If the hypothesis of normality is accepted, analysis of variance (ANOVA) will be used, followed by Tukey’s test, when necessary. The t-test for paired data will be used to analyze the results of treatment between the evaluation periods. If the hypothesis of normality is rejected, the Kruskal-Wallis test will be used, followed by the Student-Newman-Keuls test, when necessary. The Wilcoxon test will be used for the analysis of the results of each treatment between the evaluation periods. The significance level is set at α = 0.05. The future results should be available from the authors, at a reasonable request. Besides that, the authors intend to publish the results of the randomized, controlled clinical trial. Participants will also be called for a meeting to discuss results in case they are interested.

## Discussion

Halitosis can affect interpersonal relationships, exerting a negative impact on social wellbeing and quality of life. Moreover, halitosis may be related to the physical health of individuals and can have a psychological impact stemming from its role as a social barrier [21].

Studies assessing the reduction of halitosis using aPDT and probiotics are scarce. Although methylene blue and red laser have been used for this purpose, the proposed study will evaluate the effect of antimicrobial PDT with LED and an urucum on the reduction in halitosis. aPDT can be performed with an urucum (*Bixa orellana*) extract photosensitizer and LED, which is a light source that is more accessible to dentists. The fact that the urucum is red facilitates the work combined with LED and enables its use on a larger scale, as the majority of dentists already have blue LEDs at their offices. The protocol with urucum and LED has already been used and shown good clinical results in the immediate reduction of halitosis [29].

The use of probiotics in dentistry constitutes an innovative treatment modality, capable of modifying the oral microbiota, which is complex and poses a considerable challenge to the development of protocols for the prevention and treatment of halitosis.

## Supporting information

S1 File SPIRIT checklist. (DOC)

S2 File Feedback from the Ethics Committee in original language. (PDF)

S3 File Feedback from the Ethics Committee in English. (PDF)

S4 File Study protocol in original language. (DOCX)

S5 File Study protocol in English. (DOCX)
