# Supplementary material for: Comparative study between photodynamic therapy with urucum + Led and probiotics in halitosis reduction–protocol for a controlled clinical trial
Source: PLoS One. 2021 May 14;16(5):e0247096. doi: 10.1371/journal.pone.0247096 (PMC8121297; doi:10.1371/journal.pone.0247096)
Supplement: S2 File — (PDF) [file pone.0247096.s002.pdf]

PARECER CONSUBSTANCIADO DO CEP

DADOS DO PROJETO DE PESQUISA

Título da Pesquisa: ESTUDO COMPARATIVO ENTRE A TERAPIA FOTODINÂMICA COM URUCUM E LED E PROBIÓTICOS NA REDUÇÃO DA HALITOSE - ENSAIO CLÍNICO CONTROLADO RANDOMIZADO

Pesquisador: Sandra Kalil Bussadori

Área Temática:

Versão: 2

CAAE: 20123519.4.0000.5511

Instituição Proponente: ASSOCIACAO EDUCACIONAL NOVE DE JULHO

Patrocinador Principal: Financiamento Próprio

DADOS DO PARECER

Número do Parecer: 3.669.442

Apresentação do Projeto:

As informações contidas nos campos Apresentação do Projeto, Objetivo da Pesquisa e Avaliação dos Riscos e Benefícios foram extraídas do documento: PB_INFORMAÇÕES_BÁSICAS_DO_PROJETO_1403207 na data de 08_10_2019.

Halitose é um termo que define qualquer odor ou mau-cheiro proveniente da cavidade oral, que pode apresentar origem local ou sistêmica. Este projeto tem como objetivo verificar se o tratamento com terapia fotodinâmica antimicrobiana (aPDT) e o tratamento com uso de probióticos são eficazes contra esta. Serão selecionados 52 alunos ou funcionários da UNINOVE, de 18 a 25 anos, com diagnóstico de halitose, apresentando na cromatografia gasosa o sulfidreto (SH2) 112 ppb. Os participantes serão divididos aleatoriamente em 4 grupos de 13, que receberão tratamentos distintos: Grupo 1: tratamento com escovação, fio dental e raspador de língua; Grupo 2: escovação, fio dental e aPDT aplicada na região de dorso e terço médio da língua; Grupo 3:escovação fio dental e probióticos; Grupo 4: escovação, fio dental, aPDT e probióticos. Serão comparados os resultados da halimetria antes, imediatamente após o tratamento, sete dias após e trinta dias após o tratamento.

Metodologia Proposta:

Serão selecionados 52 alunos ou funcionários da UNINOVE com diagnóstico de halitose, apresentando na cromatografia gasosa o sulfidreto (SH2) 112 ppb. Os participantes serão divididos por meio de randomização em blocos em quatro grupos (n=13), de acordo com o tratamento a ser realizado (Figura 1). Grupo 1: tratamento com escovação, fio dental e raspador de língua; Grupo 2: escovação, fio dental e aPDT aplicada na região de dorso e terço médio da língua; Grupo 3: escovação fio dental e probióticos; Grupo 4: escovação, fio dental, aPDT e probióticos. Serão comparados os resultados da halimetria antes, imediatamente após o tratamento, sete dias após e trinta dias após o tratamento. Será realizada a análise microbiológica da saburra lingual nestes mesmos tempos. A análise quantitativa será realizada por meio de PCR em tempo real. Esse trabalho será enviado ao Comitê de Ética em Pesquisa da UNINOVE e por se tratar de um estudo clínico randomizado e buscando uma maior transparência e qualidade dessa pesquisa, utilizaremos as recomendações do CONSORT (Consolidated Standards of Reporting Trials).

Critério de Inclusão:
Serão incluídos nesta pesquisa participantes de ambos os sexos, de 18 a 25 anos, com diagnóstico de halitose apresentando na cromatografia gasosa o sulfidreto (SH2) 112 ppb.

Critério de Exclusão:
Serão excluídos do estudo indivíduos com anomalias dentofaciais (como lábio leporino, fissuras palatinas e nasopalatinas), em tratamento ortodôntico e/ou ortopédico, que estejam em tratamento oncológico, com alterações sistêmicas (gastrointestinais, renais, hepáticas), em tratamento com antibiótico até 1 mês antes da pesquisa e grávidas.

Objetivo da Pesquisa:
Objetivo Primário:
O objetivo do presente estudo é verificar se o tratamento com aPDT, utilizando o urucum como fotossensibilizador e o LED como fonte de luz, é
eficaz na redução imediata da halitose quando avaliada pela cromatografia gasosa, assim como comparar esse método com a utilização do raspador
lingual, método convencional mais utilizado, uso de fio dental e escovação com dentifrício com fluoreto de amina (Elmex®) e uso de probióticos
Objetivo Secundário:
Realizar a análise microbiológica quantitativa das bactérias presentes na saburra lingual antes e depois do tratamento, por meio do PCR em tempo real.

Avaliação dos Riscos e Benefícios:
Benefícios:
Sensibilidade na língua

Benefícios:
Redução da halitose

Comentários e Considerações sobre a Pesquisa:
Trata-se da segunda versão de um projeto já avaliado pelo presente Comitê de Ética.

Estudo com Delineamento controlado e randomizado que incluirá 52 participantes, 13 em cada um dos 4 grupos de tratamento:
Grupo 1: tratamento com escovação, fio dental e raspador de língua (tratamento convencional);
Grupo 2: escovação, fio dental e terapia fotodinâmica aplicada na região de dorso e terço médio da língua;
Grupo 3:escovação fio dental e probióticos;
Grupo 4: escovação, fio dental, terapia fotodinâmica e probióticos.

Considerações sobre os Termos de apresentação obrigatória:
Abaixo a lista de pendências identificadas no parecer anterior seguido dos critério: PENDÊNCIA ATENDIDA e PENDÊNCIA NÃO ATENDIDA.

1) Apresentar nova folha de rosto, incluindo o carimbo do diretor: PENDÊNCIA ATENDIDA.

Ajustar o TCLE quanto:
2) à linguagem: explicando termos técnicos: PENDÊNCIA ATENDIDA.

3) ao detalhamento no item procedimentos da fase experimental todos os procedimentos aos quais o participante será submetido: PENDÊNCIA ATENDIDA.

4) à clareza ao participante que ele poderá ser enquadrado em um dos quatro grupos do estudo e que isso se dará por sorteio: PENDÊNCIA ATENDIDA.

5) aos riscos nos procedimentos com PDT, uso de probióticos e em relação ao constrangimento: PENDÊNCIA PARCIALMENTE ATENDIDA! Essas informações aparecem no TCLE mas não no

documento: PB_INFORMAÇÕES_BÁSICAS_DO_PROJETO_1403207.

6) às medidas protetivas. Em quais situações (riscos, item anterior) pode haver necessidade de uso de medicação? Se houver risco de constrangimento, quais as medidas protetivas?: PENDÊNCIA ATENDIDA.

7) à retirada do consentimento: uma vez que participantes podem ser alunos, é importante informar que no caso de desistir de participar do estudo o aluno não terá nenhum prejuízo em relação às suas atividades acadêmicas na universidade.: PENDÊNCIA ATENDIDA.

8) No projeto, informar quanto à seleção dos participantes: PENDÊNCIA ATENDIDA.

Recomendações:
Sugere-se que na submissão das notificações semestrais, o item "riscos" quando há a submissão dos dados do projeto na Plataforma Brasil seja o mesmo do TCLE.

Conclusões ou Pendências e Lista de Inadequações:
Todas as pendências anteriores foram sanadas. Projeto Aprovado.

Tendo em vista a legislação vigente devem ser encaminhados para o CEP-UNINOVE relatórios parciais anuais referentes ao andamento da pesquisa e relatório final utilizando-se a opção “Enviar notificação” disponível na área em que encontra o seu projeto de pesquisa aprovado no campo “lista de apreciações do projeto” mais especificamente na coluna “ações”. Qualquer alteração no projeto original aprovado pode ser apresentada com “emenda” desde que não contenha modificação essencial nos objetivos e na metodologia do projeto origina. De forma objetiva com justificativa para nova apreciação e os documentos alterados devem ser evidenciados para facilitar a nova análise.

Considerações Finais a critério do CEP:
O pesquisador deverá se apresentar na instituição de realização da pesquisa (que autorizou a realização do estudo) para início da coleta dos dados.
O participante da pesquisa (ou seu representante) e o pesquisador responsável deverão rubricar todas as folhas do Termo de Consentimento Livre e Esclarecido - TCLE apondo sua assinatura na última página do referido Termo, conforme Carta Circular no 003/2011 da CONEP/CNS.
Salientamos que o pesquisador deve desenvolver a pesquisa conforme delineada no protocolo

aprovado.

Eventuais modificações ou emendas ao protocolo devem ser apresentadas ao CEP de forma clara e sucinta, identificando a parte do protocolo a ser modificada e suas justificativas. Lembramos que esta modificação necessitará de aprovação ética do CEP antes de ser implementada.

Ao pesquisador cabe manter em arquivo, sob sua guarda, por 5 anos, os dados da pesquisa, contendo fichas individuais e todos os demais documentos recomendados pelo CEP (Res. CNS 466/12 item X1. 2. f).

De acordo com a Res. CNS 466/12, X.3.b), o pesquisador deve apresentar a este CEP/SMS os relatórios semestrais. O relatório final deverá ser enviado através da Plataforma Brasil, ícone Notificação. Uma cópia digital (CD/DVD) do projeto finalizado deverá ser enviada à instância que autorizou a realização do estudo, via correio ou entregue pessoalmente, logo que o mesmo estiver concluído.

Este parecer foi elaborado baseado nos documentos abaixo relacionados:

Tipo Documento	Arquivo	Postagem	Autor	Situação
Informações Básicas do Projeto	PB_INFORMAÇÕES_BÁSICAS_DO_PROJETO_1403207.pdf	08/10/2019 16:26:11		Aceito
TCLE / Termos de Assentimento / Justificativa de Ausência	TCLE_halitose.docx	08/10/2019 16:25:54	Sandra Kalil Bussadori	Aceito
Projeto Detalhado / Brochura Investigador	Halitose_Projeto.docx	08/10/2019 16:25:42	Sandra Kalil Bussadori	Aceito
Folha de Rosto	folha_de_rosto.pdf	08/10/2019 16:25:21	Sandra Kalil Bussadori	Aceito

Situação do Parecer:
Aprovado

Necessita Apreciação da CONEP:
Não

SAO PAULO, 29 de Outubro de 2019

Assinado por:
CHRISTIANE PAVANI
(Coordenador(a))
