# Supplementary material for: Comparative study between photodynamic therapy with urucum + Led and probiotics in halitosis reduction–protocol for a controlled clinical trial
Source: PLoS One. 2021 May 14;16(5):e0247096. doi: 10.1371/journal.pone.0247096 (PMC8121297; doi:10.1371/journal.pone.0247096)
Supplement: S3 File — (PDF) [file pone.0247096.s003.pdf]

Nove de Julho University

Feedback from the Ethics Committee

RESEARCH PROJECT DATA

Research Title: COMPARATIVE STUDY BETWEEN PHOTODYNAMIC THERAPY WITH URUCUM AND LED AND PROBIOTICS IN THE REDUCTION OF HALITOSIS - RANDOMIZED CONTROLLED CLINICAL TRIAL
Researcher: Sandra Kalil Bussadori
Thematic Area:
Version: 2
Certificate of Presentation to the Ethics Committee: 20123519.4.0000.5511
Proponent institution: Nove de Julho Association
Main Sponsor: Nove de Julho Association

FEEDBACK DATA

Number: 3.669.442

Presentation of the Project:
The information contained in thePresentation of the Project, Research Objective and Risk and Benefit Assessment fields were extracted from the Document: PB_INFORMAÇÕES_BÁSICAS_DO_PROJETO_1403207 on 08_10_2019.

Halitosis is a term that defines any odor or foul smell from the oral cavity, which may be local or systemic in origin. This project aims to verify whether treatment with antimicrobial photodynamic therapy (aPDT) and treatment with the use of probiotics is effective against it. 52 UNINOVE students or employees, 18 to 25 years old, diagnosed with halitosis will be selected, presenting 112 ppb sulfide (SH2) in gas chromatography. Participants will be randomly divided into 4 groups of 13, which will receive different treatments: Group 1: treatment with teeth brushing, dental flossing and tongue scraper; Group 2: teeth brushing, dental flossing and aPDT applied to the back and middle thirds of the tongue; Group 3: teeth brushing, dental flossing and probiotics; Group 4: teeth brushing, dental flossing, aPDT and probiotics. The results of halimetry will be compared before, immediately after treatment, seven days after and thirty days after treatment.

Proposed Methodology:

52 UNINOVE students or employees diagnosed with halitosis will be selected, presenting 112ppb sulfide (SH2) in gas chromatography. Participants will be divided by block randomization into four groups (n = 13), according to the treatment to be performed (Figure 1). Group 1: treatment with teeth brushing, dental flossing and tongue scraper; Group 2: teeth brushing, dental flossing and aPDT applied to the back and middle thirds of the tongue; Group 3: teeth brushing, dental flossing and probiotics; Group 4: teeth brushing, dental flossing, aPDT and probiotics. The results of halimetry will be compared before, immediately after treatment, seven days after and thirty days after treatment. The microbiological analysis of the tongue coating will be carried out at these same times. Quantitative analysis will be performed using real-time PCR. This research will be sent to the Research Ethics Committee of UNINOVE and because it is a randomized clinical study and seeking greater transparency and quality of this research, we will use the recommendations of CONSORT (Consolidated Standards of Reporting Trials).

Inclusion Criteria:
This research will include participants of both genders, 18 to 25 years old, diagnosed with halitosis presenting gas chromatography with sulfide (SH2) 112ppb.

Exclusion Criteria:
Individuals with dentofacial anomalies (such as cleft lip, cleft palates and nasopalatine clefts) will be excluded from the study, undergoing orthodontic and/or orthopedic treatment, who are undergoing cancer treatment, with systemic changes (gastrointestinal, renal, hepatic), being treated with antibiotics up to 1 month before the survey and pregnant.

Objective of the research:

Primary Objective:
The aim of the present study is to verify whether aPDT treatment, using annatto as a photosensitizer and LED as a light source, is
effective in the immediate reduction of halitosis when evaluated by gas chromatography, as well as comparing this method with the use of the scraper
lingual, most commonly used conventional method, flossing and toothbrushing with amine fluoride (Elmex®) and use of probiotics

Secondary Objective:
Perform the quantitative microbiological analysis of bacteria present in the lingual coating before and after treatment, using real-time PCR.

Evaluation of Risks and Benefits:

Risks: Sensitivity in the tongue
Benefits: Halitosis reduction

Comments and Considerations about the Research:

This is the second version of a project already evaluated by this Ethics Committee.
Study with controlled and randomized design that will include 52 participants, 13 in each of the 4 treatment groups:
Group 1: treatment with brushing, dental floss and tongue scraper (conventional treatment);
Group 2: brushing, dental floss and photodynamic therapy applied to the back and middle third of the tongue;
Group 3: brushing dental floss and probiotics;
Group 4: brushing, dental floss, photodynamic therapy and probiotics.

Considerations about the Terms of Mandatory Presentation:

Below is the list of backlogs identified in the previous opinion followed by the criteria: ATTENDED PENDENCE and NON ATTENDED PENDENCE.

1) Submit a new cover page, including the director's stamp: ATTENDED PENDENCE.
Adjust the following items in the Consent Term:
2) language: explaining technical terms: ATTENDED PENDENCE.
3) detail the procedures in the experimental phase item, all the procedures to which the participant will be submitted: ATTENDED PENDENCE.
4) clarity to the participant that he/she may be included in one of the four study groups: ATTENDED PENDENCE.
5) the risks in the procedures with PDT, use of probiotics and in relation to the constraint: PARTIALLY ATTENDED PENDENCE! This information appears in the Consent Term but not in the PB_INFORMAÇÕES_BÁSICAS_DO_PROJETO_1403207 document.
6) protective measures. In what situations (risks, previous item) there may be a need to use medication? If there is a risk of embarrassment, what are the protective measures ?: ATTENDED PENDENCE.
7) withdrawal of consent: since participants can be students, it is important to inform that in case of giving up participating in the study, the student will have no prejudice in relation to his academic activities at the university .: ATTENDED PENDENCE.
8) In the project, inform about the selection of participants: ATTENDED PENDENCE.

Recommendations:
It is suggested that when submitting semi-annual notifications, the item "risks" when submitting project data to Plataforma Brasil is the same as it is in the Consent Term.

Conclusions or Pendencies and Inadequacies list:
All previous issues were solved. Approved Project.
In view of the current legislation, partial annual reports regarding the progress of the research and final reports should be sent to CEP-UNINOVE using the option “Send notification” available in the area where you find your approved research project in the field “list project appraisals ”more specifically in the“ actions ”column. Any changes to the original approved project can be submitted as an “amendment” as long as it does not contain an essential change in the original project objectives and methodology. In an objective way with justification for a new appraisal and the altered documents must be evidenced to facilitate the new analysis.

Concluding Remarks at the discretion of the Ethics Committee:
The researcher must presente him/herself to the institution where the research was carried out (which authorized the study) to begin data collection.
The research participant (or his representative) and the responsible researcher must sign all the Free and Informed Consent Form (TCLE) sheets, placing their signature as well on the last page of the said Term, according to Circular Letter 003/2011 of CONEP/ CNS.
We emphasize that the researcher must develop the research as outlined in the approved protocol.
Any modifications or amendments to the protocol must be presented to the CEP in a clear and succinct manner, identifying the part of the protocol to be modified and its justifications. Please note that this modification will require ethical approval from the CEP before it can be implemented.
The researcher is responsible for keeping the research data on file for 5 years, containing individual files and all other documents recommended by the CEP (Res. CNS 466/12 item X1. 2. f). According to Res. CNS 466/12, X.3.b), the researcher must submit to this CEP/SMS the semiannual reports. The final report should be sent through the Brazil Platform, icon Notification. A digital copy (CD/DVD) of the finalized project must be sent to the instance that authorized the study to be carried out, by post or delivered in person, as soon as it is completed.

This feedback has been prepared based on the documents listed below:

Type of Document	File	Postage	Author	Situation
Basic Project Information	PB_INFORMAÇÕES_BÁSICAS_DO_PROJETO_1403207.pdf	08/10/2019 16:26:11		Accepted
Consent Term/ Terms of assent / Justification of Absence	TCLE_halitose.docx	08/10/2019 16:25:54	Sandra Kalil Bussadori	Accepted
Detailed project / Investigator brochure	Halitose_Projeto.docx	08/10/2019 16:25:42	Sandra Kalil Bussadori	Accepted
Title Page	folha_de_rosto.pdf	08/10/2019 16:25:21	Sandra Kalil Bussadori	Accepted

Feedback situation:
Approved.

Is the approval from the CONEP necessary?
No.

SAO PAULO, OCTOBER 29, 2019.
Signed by:

CHRISTIANE PAVANI
(Coordinator)
