# Supplementary material for: Comparative study between photodynamic therapy with urucum + Led and probiotics in halitosis reduction–protocol for a controlled clinical trial
Source: PLoS One. 2021 May 14;16(5):e0247096. doi: 10.1371/journal.pone.0247096 (PMC8121297; doi:10.1371/journal.pone.0247096)
Supplement: S4 File — (DOCX) [file pone.0247096.s004.docx]

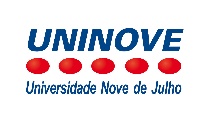


**UNIVERSIDADE NOVE DE JULHO**

**PROGRAMA DE PÓS-GRADUAÇÃO EM BIOFOTÔNICA APLICADA ÀS CIÊNCIAS DA SAÚDE**

**Pamella de Barros Motta**

**ESTUDO COMPARATIVO ENTRE A TERAPIA FOTODINÂMICA COM URUCUM E LED E PROBIÓTICOS NA REDUÇÃO DA HALITOSE - ENSAIO CLÍNICO CONTROLADO RANDOMIZADO**

**São Paulo, SP**

**2019**

**RESUMO**

Halitose é um termo que define qualquer odor ou mau-cheiro proveniente da cavidade oral, que pode apresentar origem local ou sistêmica. Este projeto tem como objetivo verificar se o tratamento com terapia fotodinâmica antimicrobiana (aPDT) e o tratamento com uso de probióticos são eficazes contra esta. Serão selecionados 52 alunos ou funcionários da UNINOVE, de 18 a 25 anos, com diagnóstico de halitose, apresentando na cromatografia gasosa o sulfidreto (SH_2_) ≥ 112 ppb. Os participantes serão divididos aleatoriamente em 4 grupos de 13, que receberão tratamentos distintos: Grupo 1: tratamento com escovação, fio dental e raspador de língua; Grupo 2: escovação, fio dental e aPDT aplicada na região de dorso e terço médio da língua; Grupo 3:escovação fio dental e probióticos; Grupo 4: escovação, fio dental, aPDT e probióticos. Serão comparados os resultados da halimetria antes, imediatamente após o tratamento, sete dias após e trinta dias após o tratamento. Será realizada a análise microbiológica da saburra lingual nestes mesmos tempos. A análise quantitativa será realizada por meio de PCR em tempo real. A normalidade dos dados será aferida através do teste Shapiro-Wilk, e, no caso de normalidade será aplicado o teste de Análise de Variância (ANOVA), e, no caso de dados não-paramétricos, será utilizado o teste Kruskal-Wallis. Para analisar os resultados de cada tratamento nos dois períodos do estudo será utilizado o teste de Wilcoxon.

**Descritores:** Halitose, Terapia Fotodinâmica, *Bixa orellana*, Probióticos

**Clinical Trials:** NCT03996044

**1. INTRODUÇÃO**

Halitose é um termo que define qualquer odor ou mau-cheiro proveniente da cavidade oral, que pode apresentar origem local ou sistêmica [1].

Compostos Sulfurosos Voláteis (CVS) são componentes químicos que estão relacionados com a halitose são eles: o sulfeto de hidrogênio (H2S), metilmercaptanas (CH3SH) e o Dimetilsulfeto (CH3SCH3) [2-5].

Existem diferentes métodos de diagnóstico de halitose: a avaliação clínica, conhecida como teste organoléptico, um método subjetivo que consiste em sentir o cheiro exalado pela boca e pelo nariz, e em seguida, quantificar esse odor com o uso de uma escala. Os CVS podem ser medidos com o uso de monitores de sulfeto. E a cromatografia gasosa, o método mais apropriado para o diagnóstico da halitose de qualquer origem, pois realiza a mensuração de 3 principais gases sulfidreto, metilmercaptana e dimetilsulfeto [6-8]. Apesar de ter uma etiologia complexa, as bactérias anaeróbias são apontadas como a principal causa [6]. A prevalência de halitose é alta, sendo possível encontrar na literatura valores acima de 50% [9].

Os tratamentos convencionais utilizados no controle da halitose consistem basicamente no uso de dentifrícios e colutórios contendo substâncias bactericidas, uso de raspador lingual, tratamento das lesões de cárie e da doença periodontal, além do controle da xerostomia [10]. Alguns estudos sugerem que o fluoreto de amina tem efeito positivo na diminuição da halitose [9].

Estudos mostram que tratamentos alternativos, como a Terapia Fotodinâmica Antimicrobiana (aPDT) [12, 13, 14] e probióticos, tem sido usados na tentativa de controlar a halitose [9, 10, 15].

A aPDT é um tratamento no qual é utilizado um agente fotossensibilizador que na presença de luz, produz radicais livres de oxigênio levando à morte celular [16].

Os probióticos são definidos como microorganismos que proporcionam efeitos benéficos para a saúde do hospedeiro quando absorvidos pelo mesmo. São frequentemente utilizados em alimentos e produtos fermentados, além de serem utilizados em manipulações farmacêuticas [17].

As vantagens de abordagens alternativas são reduzir os danos aos tecidos e evitar resistência bacteriana.

1. **2. JUSTIFICATIVA**

A halitose é considerada um importante fator social, pois interfere nas relações interpessoais. Além de gerar preocupações relacionadas à saude física do indivíduo pode provocar alterações psicológicas, conduzindo à uma barreira social [18].

Justifica-se a realização do projeto devido à escassez de estudos que avaliem a redução da halitose por meio da terapia fotodinâmica e do uso de probióticos, apresentando o urucum como fotossensibilizador e o LED como uma fonte de luz mais acessível aos dentistas. Embora o azul de metileno combinado com o laser vermelho já tenha sido utilizado com essa finalidade, este estudo irá avaliar o efeito da aPDT com urucum e LED para diminuição da halitose. O fato de que o urucum é vermelho facilita o trabalho combinado com um diodo emissor de luz (LED) e permite seu uso em uma escala maior. Além disso, o LED é de baixo custo e a maioria dos dentistas já tem o dispositivo em seus consultórios. Protocolos com o uso de LED na redução da halitose já foram desenvolvidos e obtiveram resultados positivos, observando recolonização a partir de 7 dias do tratamento [12, 19], a proposta desse projeto é dar continuidade a esses protocolos, acrescentando o uso de probióticos e a análise por cromatografia gasosa até 30 dias após o tratamento para avaliar sua eficácia.

O uso de probióticos na odontologia apresenta um tratamento inovador, capaz de modificar a microbiota oral, principalmente no que diz respeito à halitose, já que a microbiota oral é complexa sendo um grande desafio no desenvolvimento de protocolos de prevenção e tratamento da doença [20].

O presente estudo propõe um ensaio clínico controlado para comparar o efeito da terapia fotodinâmica e o uso de probióticos no controle da halitose.

1. **3. Hipóteses**

*Hipótese experimental:* Há diminuição da halitose após o uso da terapia fotodinâmica empregando o uso de corante azul e LED vermelho. Há diminuição da halitose após o tratamento com probióticos. Há alteração microbiológica após a terapia fotodinâmica antimicrobiana. Há alteração microbiológica após o tratamento com probióticos.

*Hipótese nula:* Não há alteração da halitose após o uso da terapia fotodinâmica empregando o uso de corante azul e LED vermelho. Não há alteração da halitose após o tratamento com probióticos Não há alteração microbiológica após a terapia fotodinâmica antimicrobiana. Não há alteração microbiológica após o tratamento com probióticos.

1. **4. OBJETIVO**

O objetivo do presente estudo é verificar se o tratamento com aPDT, utilizando o urucum como fotossensibilizador e o LED como fonte de luz, é eficaz na redução imediata da halitose quando avaliada pela cromatografia gasosa, assim como comparar esse método com a utilização do raspador lingual, método convencional mais utilizado, uso de fio dental e escovação com dentifrício com fluoreto de amina (Elmex®) e uso de probióticos.

Realizar a análise microbiológica quantitativa das bactérias presentes na saburra lingual antes e depois do tratamento, por meio do PCR em tempo real.

1. **MATERIAL E MÉTODOS**

Será realizado um convite e os participantes que se interessarem em participar serão selecionados. Serão incluídos 52 alunos ou funcionários da UNINOVE com diagnóstico de halitose, apresentando na cromatografia gasosa o sulfidreto (SH_2_) ≥ 112 ppb. Os participantes serão divididos por meio de randomização em blocos em quatro grupos (n=13), de acordo com o tratamento a ser realizado (Figura 1). Grupo 1: tratamento com escovação, fio dental e raspador de língua; Grupo 2: escovação, fio dental e aPDT aplicada na região de dorso e terço médio da língua; Grupo 3: escovação fio dental e probióticos; Grupo 4: escovação, fio dental, aPDT e probióticos. Serão comparados os resultados da halimetria antes, imediatamente após o tratamento, sete dias após e trinta dias após o tratamento. Será realizada a análise microbiológica da saburra lingual nestes mesmos tempos. A análise quantitativa será realizada por meio de PCR em tempo real. Esse trabalho será enviado ao Comitê de Ética em Pesquisa da UNINOVE e por se tratar de um estudo clínico randomizado e buscando uma maior transparência e qualidade dessa pesquisa, utilizaremos as recomendações do CONSORT (Consolidated Standards of Reporting Trials)

- 1. **Critérios de Inclusão**

Serão incluídos nesta pesquisa participantes de ambos os sexos, de 18 a 25 anos, com diagnóstico de halitose apresentando na cromatografia gasosa o sulfidreto (SH_2_) ≥ 112 ppb.

- 1. **Critérios de Exclusão**

Serão excluídos do estudo indivíduos com anomalias dentofaciais (como lábio leporino, fissuras palatinas e nasopalatinas), em tratamento ortodôntico e/ou ortopédico, que estejam em tratamento oncológico, com alterações sistêmicas (gastrointestinais, renais, hepáticas), em tratamento com antibiótico até 1 mês antes da pesquisa e grávidas.

**Randomização**

A randomização será realizada utilizando o site randomizer.org e a ordem será colocada em envelope pardo e o participante irá retirar o envelope no momento do tratamento.

• **Procedimentos**

Por se tratar de um estudo clínico randomizado e buscando uma maior transparência e qualidade dessa pesquisa, utilizaremos as recomendações CONSORT (Consolidated Standards of Reporting Trials) (figura 1).

**Indivíduos da clínica odontológica da UNINOVE**

**Excluídos**

**Recrutamento**

**Halimetria**

**Excluídos SH2<112ppb**

**Diagnóstico SH2>112ppb**

**n=52**

**Grupo 4**

**n=13**

**Grupo 3**

**n=13**

**Grupo 2**

**n=13**

**Grupo 1**

**n=13**

**Escovação, fio dental, aPDT e probióticos**

**Escovação, fio dental e probióticos**

**Escovação, fio dental e raspador**

**Escovação, fio dental e Tratamento com aPDT**

**Nova halimetria imediatamente após o tratamento**

**Halimetria após 7 dias**

**Halimetria após 30 dias**

**Análise dos resultados**

**Figura 1**: Fluxograma de atividades.

1. **INTERVENÇÕES**

**Grupo 1 - Escovação, fio dental e raspador**

1. Halimetria inicial
2. Coleta de saburra lingual com swab
3. Raspagem lingual
4. Orientações de Higiene Oral
5. Halimetria imetiatamente após raspagem
6. Halimetria após 7 e 30 dias

**Grupo 2 – Escovação, fio dental e aPDT**

1. Halimetria inicial
2. Coleta de saburra lingual com swab
3. Aplicação de spray de urucum no dorso da língua por 2 minutos
4. Aplicação de LED em 6 pontos
5. Orientações de Higiene Oral
6. Halimetria imetiatamente após aPDT
7. Halimetria após 7 e 30 dias

**Grupo 3 – Escovação fio dental e uso de probióticos**

1. Halimetria inicial
2. Coleta de saburra lingual com swab
3. Entrega e orientações quanto ao uso dos probióticos
4. Orientações de Higiene Oral
5. Halimetria no 14º dia de tratamento com probióticos
6. Halimetria 7 e 30 dias após a finalização do tratamento

**Grupo 4- Escovação, fio dental, aPDT e probióticos**

1. Halimetria inicial
2. Coleta de saburra lingual com swab
3. Aplicação de spray de urucum no dorso da língua por 2 minutos
4. Aplicação de LED em 6 pontos
5. Entrega e orientações quanto ao uso dos probióticos
6. Orientações de Higiene Oral
7. Halimetria no 14º dia de tratamento com probióticos
8. Halimetria 7 e 30 dias após a finalização do tratamento

**Cálculo de amostra**

Para o cálculo do tamanho amostral foram utilizados os dados do trabalho de Costa da Mota *et al*. (Effect of photodynamic therapy for the treatment of halitosis in adolescents - a controlled, microbiological, clinical trial).

Inicialmente estabeleceu-se um erro $err=\left| \bar{x_{1}}-\bar{x_{2}} \right|$, onde$\bar{x_{1}}$ e $\bar{x_{2}}$ sãos os valores médios dos grupos baseline a tratamento periodontal com PDT. A partir deste erro, foi calculado o effect size, dado por

$$\frac{err}{\sqrt{\sigma_{1}^{2}+\sigma_{2}^{2}}}$$

onde $\sigma_{1}^{2}$e $\sigma_{2}^{2}$ são as variâncias dos grupos um e dois, respectivamente.

Assumindo que os grupos estudados possuem distribuição normal ou aproximadamente normal, que o tamanho amostral será suficientemente grande e que será utilizado um teste bicaudal, para um nível de significância α = 0,05 e mantendo o poder do teste 1-β = 0,90, temos um n=13 para cada grupo.

Na figura 3 observa-se que com um tamanho amostral total de 39 sujeitos, ou seja, três grupos com 13 amostras cada, que deverá ser demonstrada a diferença estatística mantendo o poder do teste maior ou igual a 0.90. Caso a hipótese de normalidade das distribuições seja rejeitada, o tamanho amostral deverá ser corrigido em aproximadamente 5%.

Figura 2: Ajuste do poder do teste em função do tamanho amostral total.

Na figura 2 observa-se que com um tamanho amostral total de 52 sujeitos, ou seja, quatro grupos com 13 amostras cada, que deverá ser demonstrada a diferença estatística mantendo o poder do teste maior ou igual a 0.90. Caso a hipótese de normalidade das distribuições seja rejeitada, o tamanho amostral deverá ser corrigido em aproximadamente 5%.

*Halimetria*

A coleta do ar bucal seguirá as orientações do fabricante (Oral Chroma^TM^ Manual Instruction), onde o participante será orientado a fazer bochecho com cisteína (10 mM) por 1 minuto, em seguida permanecer com a boca fechada mais 1 minuto. Será introduzida na boca do participante uma seringa do mesmo fabricante própria para coleta do ar bucal. Durante 1 minuto o participante permanecerá de boca fechada, respirando pelo nariz, sem tocar na seringa com a língua. O êmbolo será puxado para fora, voltaremos a esvaziar o ar da seringa na boca do participante e novamente puxaremos o êmbolo para encher a seringa com a amostra do hálito. Iremos limpar a ponta da seringa com gaze para remover a umidade da saliva, colocar a agulha de injeção de gás na seringa, e ajustar o êmbolo para 0,5 ml. Injetam-se os gases coletados na porta de entrada do aparelho com um movimento único (Figura 4).

**Figura 4:** Processo de realização da halimetria.

Da análise dos VSCs capturados pelo sistema, temos:

- Sulfidreto: origem principalmente das bactérias presentes no dorso da língua. Valores acima de 112 ppb são indicadores de halitose.

- Metilmercaptana: predominantemente mais elevada nas bolsas periodontais. Valores até 26 ppb são considerados normais. A doença periodontal resulta tipicamente numa alta razão entre metilmercaptana/sulfidreto (>3:1)

- Dimetilsulfeto: tanto pode ser de origem periodontal como de origem sistêmica (intestinal, hepática, pulmonar). Também pode ser causado, temporariamente, pela ingestão de certos alimentos e bebidas. Há possibilidade de se fazer a distinção entre o dimetilsulfeto de origem bucal e o de origem sistêmica, através da comparação dos resultados da halimetria com e sem o desafio da cisteína (cisteína 10 mM, ou seja, 16 mg de cisteína em 100 ml de água destilada – 16 mg%). O limiar de percepção do dimetilsulfeto é o mais baixo, 8 ppb. Outros odores (não VSCs) podem aparecer num pico anterior ao teoricamente primeiro pico que é o do sulfidreto.

Para evitar alterações na halimetria os participantes serão instruídos a seguir as seguintes orientações: 48 horas antes da avaliação evitar a ingestão de alimentos com alho, cebola e temperos fortes, consumo de álcool e uso de antisséptico bucal. No dia da avaliação, pela manhã, poderão alimentar-se até no máximo 2 horas antes do exame, abster-se de café, balas, goma de mascar, produtos de higiene oral e pessoal com perfume (pós-barba, desodorante, perfume, cremes e/ou tônico) e a escovação será apenas com água.

*Análise Microbiológica*

As amostras da saburra lingual serão coletadas utilizando 1 swab estéril que será passado na superfície do dorso de língua com movimento de vai e vem 10 vezes. As amostras serão depositadas em tubos estéreis que serão identificados e armazenados à -80 C até serem analisados. Após descongelamento, as amostras serão submetidas ao vórtex por um minuto. Para extração do DNA bacteriano as amostras serão submetidas a um banho de fervura por 10 minutos sendo posteriormente centrifugadas a 10.000 rpm por 10 minutos. O sobrenadante será colocado em um novo microtubo contendo100μL de fenol/clorofórmio/álcool isoamílico (25:24:1), seguido da precipitação com etanol. O DNA purificado será ressuspenso em tampão TE. Serão analisados os níveis de P. gingivalis, T. forsythia e T. denticola, por PCR quantitativo. A análise quantitativa será realizada por meio de PCR em tempo real utilizando termociclador Step One Plus Real-Time PCR System (Applied Biosystem, Foster City, CA, EUA) e os produtos detectados por fluorescência usando o Quantimix Easy SYG Kit (Biotools, Madri, Espanha), seguindo o protocolo recomendado pelo fabricante. Para a reação serão utilizados 10 µl de SYBR Green, 0,5 µl DNA molde, 200 mM de cada iniciador (P.gingivalis CATAGATATCACGAGGAACTCCGA TT e AAACTGTTAGCAACTACCGATGTGG; T.forsythia GGGTGAGTAACGCGTATGTAACCT e ACCCATCCGCAACCAATAAA; T. denticola CGTTCCTGGGCCTTGTACA e TAGCGACTTCAGGTACCCTCG; Universal para bactéria CCATGAAGTCGGAATCGCTAG e GCTTGACGGGCGGTGT) em volume total de 20 µl. Para a curva padrão serão realizadas reações contendo como DNA molde 2 a 2X105 cópias do gene analisado (16S rRNA) utilizando-se plasmídeos pTOPO em que serão clonados os genes 16S dos 14 diferentes organismos.  Como controle negativo será adicionada água milliQ estéril ao invés de DNA molde. As reações para 16S rRNA serão realizadas com desnaturação inicial de 95 ºC por 2 minutos, seguida de 36 ciclos de 94 ºC por 30 segundos, 55 ºC por 1 minuto e 72 ºC por 2 minutos e extensão final a 72 ºC por 10 minutos 46.  A fluorescência será detectada após cada ciclo e representada em um gráfico utilizando o software Step One Plus Real- Time PCR System (Applied Biosystem, Foster City, CA, EUA). Para garantir a especificidade dos produtos detectados por fluorescência e evitar a detecção de dímeros de iniciadores, a detecção será realizada um grau abaixo da temperatura de dissociação dos amplicons. Todas as amostras serão analisadas em duplicata e cada diluição dos plasmídeos para a curva padrão em triplicata. A finalidade da avaliação microbiológica será verificar a efetividade da terapia fotodinâmica para o tratamento da halitose, complementando a avaliação clínica.

*Terapia Fotodinâmica Antimicrobiana (aPDT)*

Será utilizado o aparelho fotopolimerizador LED – Valo Cordless Ultradent^®^, um aparelho de consultório, com radiômetro acoplado, espectro de 440-480nm e irradiância de 450mW/cm. No momento da realização da aPDT estarão presentes somente o participante a ser tratado e o profissional responsável, ambos utilizando óculos específicos para proteção ocular. A ponta ativa do LED será revestida com plástico transparente descartável (PVC) (evitando contaminações cruzada e por motivo de higiene) e o profissional estará devidamente paramentado.

Será realizada 1 sessão de aPDT com o fotossensibilizador (FS) urucum manipulado na concentração 20% (Fórmula e Ação^®^) em spray, a ser aplicado em quantidade suficiente para cobrir o terço médio e dorso da língua (5 borrifadas) por 2 minutos para incubação. O excesso será removido com sugador de forma a manter a superfície úmida com o próprio FS, sem utilização de água. Serão irradiados 6 pontos com distância de 1 cm entre os pontos, considerando o halo de espalhamento da luz e efetividade da aPDT. O aparelho estará previamente calibrado com comprimento de onda 395-480 nm, durante 20 segundos por ponto, energia de 9,6J, e a luz era irradiada de modo que seja formado um halo de 2 cm de diâmetro por ponto. A Tabela 1 contém todos os parâmetros utilizados.

Tabela 1: parâmetros do LED.

| Comprimento de onda (nm) | 395-480 |
| --- | --- |
| Modo de funcionamento | Contínuo |
| Potência radiante média (mW) | 480 |
| Polarização | aleatória |
| Diâmetro de abertura (cm) | 0.9 |
| Irradiância na abertura (mW/cm2) | 762 |
| Perfil do Feixe | Top hat |
| Área irradiada (cm2) | 3.14 |
| Irradiância no alvo (mW/cm2) | 153 |
| Tempo de exposição (s) | 20 |
| Fluência (J/cm2) | 6.37 |
| Energia radiante (J) | 9.6 |
| Número de pontos irradiados | 6 |
| Área total irradiada (cm2) | 18.8 |
| Número de sessões | 1 |
| Energia radiante total (J) | 57.6 |

*Raspagem Lingual*

A raspagem lingual será realizada por um mesmo operador em todos os participantes. Serão realizados movimentos póstero-anteriores com o raspador sobre o dorso lingual, seguidos da limpeza do raspador com uma gaze. Esse procedimento será realizado dez vezes em cada participante, com o objetivo de padronizar a remoção mecânica da saburra lingual.

*Tratamento com probióticos*

Serão utilizadas cápsulas manipuladas em farmácia contendo cepas de Lactobacillus salivarius WB21 (6,7 x 10^8^ UFC) e xilitol (280mg). Serão entregues 42 cápsulas para cada participante, que deverá ingerir 1 cápsula, 3 vezes ao dia após as refeições, durante 14 dias.

*Escovação com dentifrício com fluoreto de amina*

Todos os 52 participantes serão orientados a realizar escovação com dentifrício contendo fluoreto de amina em sua composição (Elmex®) e uso de fio dental, 3 vezes ao dia após as refeições durante 30 dias.

*Análise Estatística*

Os dados oriundos do Oral Chorma^TM^ serão analisados quanto à sua normalidade pelo teste de Shapiro – Wilk. Caso a hipótese de normalidade seja aceita, será utilizada a Análise de variância (ANOVA) seguida pelo teste de Tukey quando necessário. Para analisar os resultados do tratamento nos dois períodos do estudo será utilizado o teste T para dados pareados. Caso a hipótese de normalidade seja rejeitada, será utilizado o teste Kruskal-Wallis seguido pelo teste de Student-Newman-Keuls, quando necessário. Para analisar os resultados de cada tratamento nos dois períodos do estudo será utilizado o teste de Wilcoxon.

**4- DISCUSSÃO**

A halitose é considerada um importante fator social, pois interfere nas relações interpessoais. Além de gerar preocupações relacionadas à saude física do indivíduo pode provocar alterações psicológicas, conduzindo à uma barreira social [18].

Há uma escassez de estudos que avaliem a redução da halitose por meio da terapia fotodinâmica e do uso de probióticos, apresentando o urucum como fotossensibilizador e o LED como uma fonte de luz mais acessível aos dentistas. Embora o azul de metileno combinado com o laser vermelho já tenha sido utilizado com essa finalidade, este estudo irá avaliar o efeito da aPDT com urucum e LED para diminuição da halitose. O fato de que o urucum é vermelho facilita o trabalho combinado com um diodo emissor de luz (LED) e permite seu uso em uma escala maior. Além disso, o LED é de baixo custo e a maioria dos dentistas já tem o dispositivo em seus consultórios.

O uso de probióticos na odontologia apresenta um tratamento inovador, capaz de modificar a microbiota oral, principalmente no que diz respeito à halitose, já que a microbiota oral é complexa sendo um grande desafio no desenvolvimento de protocolos de prevenção e tratamento da doença [19].

O presente estudo propõe um ensaio clínico controlado para comparar o efeito da terapia fotodinâmica e o uso de probióticos no controle da halitose.

| ***Mês/Ano →***  ***Atividades*** | 03/19 | 04/19 | 05/19 | 06/19 | 07/19 | 08/19 | 09/19 | 10/19 | 11/19 | 12/19 | 01/20 | 02/20 | 03/20 | 04/20 | 05/20 | 06/20 | 07/20 | 08/20 | 09/20 | 10/20 | 11/20 | 12/20 | 01/21 | 02/21 |
| --- | --- | --- | --- | --- | --- | --- | --- | --- | --- | --- | --- | --- | --- | --- | --- | --- | --- | --- | --- | --- | --- | --- | --- | --- |
| ***Encaminhar para Comitê de Ética*** |  |  |  |  | X |  |  |  |  |  |  |  |  |  |  |  |  |  |  |  |  |  |  |  |
| ***Revisão***  ***da Literatura*** | X | X | X |  |  |  |  |  |  |  |  |  |  |  |  |  |  |  |  |  |  |  |  |  |
| ***Escrever Material***  ***Método*** |  |  |  | X | X | X | X | X |  |  |  |  |  |  |  |  |  |  |  |  |  |  |  |  |
| ***Triagem dos participantes*** |  |  |  |  |  |  |  |  | X | X | X | X | X | X |  |  |  |  |  |  |  |  |  |  |
| ***Execução da Metodologia*** |  |  |  |  |  |  |  |  |  |  |  |  |  |  | X |  |  |  |  |  |  |  |  |  |
| ***Análise dos Dados*** |  |  |  |  |  |  |  |  |  |  |  |  |  |  |  | X | X |  |  |  |  |  |  |  |
| ***Escrever Resultados*** |  |  |  |  |  |  |  |  |  |  |  |  |  |  |  |  |  | X | X | X |  |  |  |  |
| ***Escrever Discussão e Conclusão*** |  |  |  |  |  |  |  |  |  |  |  |  |  |  |  |  |  |  |  |  | X | X | X |  |
| ***Encaminhar Artigo para Publicação*** |  |  |  |  |  |  |  |  |  |  |  |  |  |  |  |  |  |  |  |  |  |  |  | X |

**CRONOGRAMA**

**REFERÊNCIAS**

1. ARMSTRONG, Brenda L.; SENSAT, Michelle L.; STOLTENBERG, Jill L. Halitosis: a review of current literature. **American Dental Hygienists' Association**, v. 84, n. 2, p. 65-74, 2010.
2. CALIL, CM.; MARCONDES, FK. Influence of anxiety on the production of oral volatile sulfur compounds. Life Science, v. 79, n. 7, p. 660–4, 10 jul. 2006.
3. SPRINGFIELD, J. et al. Spontaneous fluctuations in the concentrations of oral sulfurcontaining gases. J Dental Res, v. 80, n. 5, p. 1441–1444, 2001.
4. TANGERMAN, A; WINKEL, E. G. The portable gas chromatograph OralchromaTM: a method of choice to detect oral and extra-oral halitosis. J Breath Res, v. 2, n. 1, mar. 2008.
5. TOLENTINO, E. D. S.; CHINELLATO, L. E. M.; TARZIA, O. Saliva and tongue coating pH before and after use of mouthwashes and relationship with parameters of halitosis. J Appl Oral Sci, v. 19, n. 2, p. 90–4, abr. 2011.
6. PORTER, S. R.; SCULLY, C. Oral malodour (halitosis). Bmj, v. 333, n. 7569, p. 632-635, 2006.
7. KARA, C. et al. Effect of Nd: YAG laser irradiation on the treatment of oral malodour associated with chronic periodontitis. Int Dent J, v. 58, p. 151–158, 2008.
8. KARA, C; TEZEL, A; ORBAK, R. Effect of oral hygiene instruction and scaling on oral malodour in a population of Turkish children with gingival inflammation. Int J Paediatr Dent, v. 16, n. 6, p. 399–404, nov. 2006.
9. BICAK, Damla Aksit. A current approach to halitosis and oral malodor-A mini review. The open dentistry journal, v. 12, p. 322, 2018.
10. SUZUKI, Nao et al. Induction and Inhibition of Oral Malodor. Molecular Oral Microbiology, 2019.
11. SCULLY, C.; GREENMAN, J. Halitology (breath odour: aetiopathogenesis and management). Oral diseases, v. 18, n. 4, p. 333-345, 2012.
12. DA CIARCIA, Ana Carolina Costa et al. Action of antimicrobial photodynamic therapy with red leds in microorganisms related to halitose. Medicine, v. 98, n. 1, 2019.
13. KELLESARIAN, Sergio Varela et al. Effect of antimicrobial photodynamic therapy and laser alone as adjunct to mechanical debridement in the management of halitosis: A systematic review. Quintessence International, v. 48, n. 7, 2017.
14. COSTA DA MOTA, Ana Carolina et al. Effect of photodynamic therapy for the treatment of halitosis in adolescents–a controlled, microbiological, clinical trial. Journal of biophotonics, v. 9, n. 11-12, p. 1337-1343, 2016.
15. YOO, Jun-Il et al. The Effect of Probiotics on Halitosis: a Systematic Review and Meta-analysis. Probiotics and antimicrobial proteins, v. 11, n. 1, p. 150-157, 2019.
16. HOPE, Chris K.; WILSON, M. Induction of lethal photosensitization in biofilms using a confocal scanning laser as the excitation source. Journal of Antimicrobial Chemotherapy, v. 57, n. 6, p. 1227-1230, 2006.
17. SALMINEN, Seppo et al. Demonstration of safety of probiotics—a review. International journal of food microbiology, v. 44, n. 1-2, p. 93-106, 1998.
18. ELIAS, Marina Sá; FERRIANI, Maria das Graças Carvalho. Aspectos históricos e sociais da halitose. Revista Latino-Americana de Enfermagem, v. 14, n. 5, 2006.
19. GONÇALVES, Marcela Leticia Leal et al. Photodynamic therapy with Bixa orellana extract and LED for the reduction of halitosis: study protocol for a randomized, microbiological and clinical trial. Trials, v. 19, n. 1, p. 590, 2018.
20. MAHASNEH, Sari; MAHASNEH, Adel. Probiotics: a promising role in dental health. Dentistry journal, v. 5, n. 4, p. 26, 2017.

**TCLE - Termo de Consentimento livre e esclarecido para Participação em Pesquisa Clínica:**

Nome do participante:_____________________________________________________ Endereço:___________________________________________________ Telefone para contato:____________________Cidade:________________CEP:________

E-mail:________________________________________________________

**1.Título do Trabalho Experimental:** ESTUDO COMPARATIVO ENTRE A TERAPIA FOTODINÂMICA COM URUCUM E LED E PROBIÓTICOS NA REDUÇÃO DA HALITOSE - ENSAIO CLÍNICO CONTROLADO RANDOMIZADO

**2.Objetivo:** Avaliar se o tratamento com LED (uma luz) e um corante (terapia fotodinâmica, e uso de probióticos são eficazes no tratamento do mau hálito.

**3.Justificativa:** A justificativa dessa pesquisa é a busca de uma alternativa para o tratamento convencional, produzindo resultado mais duradouro e mais conforto durante o tratamento.

**4. Procedimentos da Fase Experimental:** O Sr(a) está sendo convidado a receber tratamento para mau hálito, utilizando o LED (uma luz) com um corante para eliminar as bactérias ou tomar probióticos (que são produtos alimentares que contem microrganismos vivos cuja ingestão traz benefícios à saúde), ou o método convencional (raspagem da língua), na clínica da Uninove.

O Sr(a) realizará uma avaliação da presença ou não do mau hálito e poderá participar de um dos quatro grupos dessa pesquisa conforme sorteio.

O grupo 1 fará uma raspagem lingual e receberá orientação de escovação, fio dental. O grupo 2 receberá um tratamento com um spray corante e uma luz azul e também orientação de escovação e fio dental. O grupo 3 receberá o tratamento com probióticos e orientação de escovação e fio dental. E o grupo 4 receberá o tratamento com um spray corante e luz azul e também probióticos, além da orientação de escovação e fio dental.

Antes e após o tratamento, será realizada a avaliação do mau hálito e a raspagem lingual. O mau hálito será avaliado novamente depois de 7 dias e 3º dias.

**5.Desconforto ou Riscos Esperados:** Os participantes podem sentir constrangimento em relação ao mau hálito. Os participantes que receberem o tratamento com corante podem apresentar sensibilidade na língua. O probiótico não apresenta risco nem efeito colateral por apresentarem microrganismos que já estão presentes no corpo.

**6. Medidas protetivas aos riscos:** A Equipe estará à disposição caso o participante apresente sensibilidade para remoção imediata do produto. Para evitar constrangimento a avaliação e o tratamento serão realizados em sala reservada com a presença apenas do participante e do pesquisador.

**7. Benefícios da Pesquisa:** Receber tratamento para mau hálito.

**8. Métodos Alternativos Existentes:** Não há.

**9. Retirada do Consentimento:** o participante tem a liberdade de retirar seu consentimento a qualquer momento e deixar de participar do estudo, sem nenhum prejuízo. Importante ressaltar que no caso de desistir de participar do estudo o sr(a) não terá nenhum prejuízo em relação às suas atividades acadêmicas ou profissionais na universidade.

**10. Garantia do Sigilo:** Os pesquisadores asseguram a privacidade dos participantes quanto aos dados confidenciais envolvidos na pesquisa.

**11. Formas de Ressarcimento das Despesas decorrentes da Participação na Pesquisa:** Não haverá ressarcimento.

**12. Local da Pesquisa:** A pesquisa será realizada na Clínica de Odontologia da Uninove, localizada na Rua Vergueiro, 235/249 – 2° subsolo - Liberdade, São Paulo - SP, 01504-001, Telefone: (11) 2633-9000.

**13. Comitê de Ética em Pesquisa (CEP)** é um colegiado interdisciplinar e independente, que deve existir nas instituições que realizam pesquisas envolvendo seres humanos no Brasil, criado para defender os interesses dos participantes de pesquisas em sua integridade e dignidade e para contribuir no desenvolvimento das pesquisas dentro dos padrões éticos (Normas e Diretrizes Regulamentadoras da Pesquisa envolvendo Seres Humanos – Res. CNS nº 466/12 e Res. CNS 510/2016). O Comitê de Ética é responsável pela avaliação e acompanhamento dos protocolos de pesquisa no que corresponde aos aspectos éticos. Endereço do Comitê de Ética da Uninove: Rua. Vergueiro nº 235/249 – 12º andar - Liberdade – São Paulo – SP CEP. 01504-001 Fone: 3385-9010 comitedeetica@uninove.br. Horário de atendimento do Comitê de Ética: segunda-feira a sexta-feira – Das 11h30 às 13h00 e Das 15h30 às 19h00

**14. Nome Completo e telefones dos Pesquisadores para Contato:** Prof. Dra Sandra Kalil Bussadori (011) 98381-7453, e aluna Pamella de Barros Motta- (011) 97397-6458.

**15. Eventuais intercorrências que vierem a surgir no decorrer da pesquisa poderão ser discutidas pelos meios próprios.**

São Paulo, de de 20__.

**16. Consentimento Pós-Informação:**

Eu, ________________________________________________, após leitura e compreensão deste termo de informação e consentimento, entendo que minha participação é voluntária, e que posso sair a qualquer momento do estudo, sem prejuízo algum. Confirmo que recebi uma via deste termo de consentimento, e autorizo a realização do trabalho de pesquisa e a divulgação dos dados obtidos somente neste estudo no meio científico.

___________________________________________________

Assinatura do Participante

(Todas as folhas devem ser rubricadas pelo participante da pesquisa)

17. Eu, ________________________________________ (Pesquisador do responsável desta pesquisa), certifico que:

a) Considerando que a ética em pesquisa implica o respeito pela dignidade humana e a proteção devida aos participantes das pesquisas científicas envolvendo seres humanos;

b) Este estudo tem mérito científico e a equipe de profissionais devidamente citados neste termo é treinada, capacitada e competente para executar os procedimentos descritos neste termo;

____________________________________

Assinatura do Pesquisador Responsável
