# Supplementary material for: Comparative study between photodynamic therapy with urucum + Led and probiotics in halitosis reduction–protocol for a controlled clinical trial
Source: PLoS One. 2021 May 14;16(5):e0247096. doi: 10.1371/journal.pone.0247096 (PMC8121297; doi:10.1371/journal.pone.0247096)
Supplement: S5 File — (DOCX) [file pone.0247096.s005.docx]

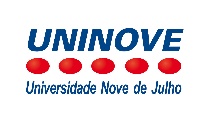


**UNIVERSIDADE NOVE DE JULHO**

**POST GRADUATION PROGRAM IN BIOPHOTONICS APPLIED TO HEALTH SCIENCES**

**Pamella de Barros Motta**

**COMPARATIVE STUDY BETWEEN PHOTODYNAMIC THERAPY WITH URUCUM AND LED AND PROBIOTICS IN THE REDUCTION OF HALITOSIS – RANDOMIZED, CONTROLLED CLINICAL TRIAL**

**São Paulo, SP**

**2019**

**ABSTRACT**

Halitosis is a term that defines any odor or foul smell that comes from the oral cavity, which can have local or systemic origin. This project aims to verify whether treatment with antimicrobial photodynamic therapy (aPDT) and treatment with the use of probiotics are effective against it. 52 UNINOVE students or employees, 18 to 25 years old, diagnosed with halitosis will be selected, presenting gas chromatography with sulfide (SH2) ≥ 112 ppb. Participants will be randomly divided into 4 groups of 13, who will receive different treatments: Group 1: treatment with teeth brushing, dental floss and tongue scraper; Group 2: teeth brushing, dental floss and aPDT applied to the back and middle thirds of the tongue; Group 3: teeth brushing, dental floss and probiotics; Group 4: teeth brushing, dental floss, aPDT and probiotics. The results of halimetry will be compared before, immediately after treatment, seven days after and thirty days after treatment. The microbiological analysis of the tongue coating will be carried out at these same times. Quantitative analysis will be performed using real-time PCR. The normality of the data will be assessed using the Shapiro-Wilk test, and, in the case of normality, the Analysis of Variance test (ANOVA) will be applied, and, in the case of non-parametric data, the Kruskal-Wallis test will be used. To analyze the results of each treatment in the two study periods, the Wilcoxon test will be used.

Keywords: Halitosis, Photodynamic Therapy, *Bixa orellana*, Probiotics

**Clinical Trials:** NCT03996044

1. **INTRODUCTION**

Halitosis is a term that defines any odor or foul smell from the oral cavity, which may be local or systemic in origin [1].

Volatile Sulfurous Compounds (VSC) are chemical components that are related to halitosis, such as: hydrogen sulfide (H2S), methylmercaptan (CH3SH) and Dimethyl sulfide (CH3SCH3) [2-5].

There are different methods for diagnosing halitosis: clinical evaluation, known as an organoleptic test, a subjective method that consists of smelling the scent exhaled by the mouth and nose, and then quantifying that odor using a scale. VSC can be measured using sulfide monitors. Gas chromatography is the most appropriate method for the diagnosis of halitosis of any origin, as it performs the measurement of the 3 main gases, sulfide, methylmercaptan and dimethylsulfide [6-8]. Despite having a complex etiology, anaerobic bacteria are identified as the main cause of halitosis [6]. The prevalence of halitosis is high, and it is possible to find values ​​above 50% in the literature [9].

The conventional treatments used to control halitosis basically consist of the use of toothpastes and mouthwashes containing bactericidal substances, use of a tongue scraper, treatment of caries lesions and periodontal disease, in addition to xerostomia control [10]. Some studies suggest that amine fluoride has a positive effect in reducing halitosis [9].

Studies show that alternative treatments, such as Antimicrobial Photodynamic Therapy (aPDT) [12, 13, 14] and probiotics, have been used in an attempt to control halitosis [9, 10, 15].

aPDT is a treatment in which a photosensitizing agent is used, and, in the presence of light, produces oxygen free radicals leading to cell death [16].

Probiotics are defined as microorganisms that provide beneficial effects on the health of the host when absorbed. They are frequently used in fermented foods and products, in addition to being used in pharmaceutical manipulations [17].

The advantages of alternative approaches include the reduction of tissue damage and avoiding bacterial resistance.

1. **2. JUSTIFICATION**

Halitosis is considered an important social factor, as it interferes with interpersonal relationships. In addition to raising concerns related to the individual's physical health, it can cause psychological changes, leading to a social barrier [18].

The project is justified due to the scarcity of studies that evaluate the reduction of halitosis through photodynamic therapy and the use of probiotics, presenting annatto as a photosensitizer and LED as a more accessible light source for dentists. Although methylene blue combined with red laser has already been used for this purpose, this study will evaluate the effect of aPDT with annatto and LED to reduce halitosis. The fact that the annatto is red facilitates work combined with a light-emitting diode (LED) and allows its use on a larger scale. In addition, the LED is inexpensive and most dentists already have the device in their offices. Protocols with the use of LEDs to reduce halitosis have already been developed and obtained positive results, observing recolonization after 7 days of treatment [12, 19]. The purpose of this project is to continue these protocols, adding the use of probiotics and gas chromatography analysis up to 30 days after treatment to assess its effectiveness.

The use of probiotics in dentistry presents an innovative treatment, capable of modifying the oral microbiota, especially with regard to halitosis, since the oral microbiota is complex, being a great challenge in the development of disease prevention and treatment protocols [20].

The present study proposes a controlled clinical trial to compare the effect of photodynamic therapy and the use of probiotics to control halitosis.

1. **3. HYPOTHESIS**

*Experimental hypothesis*: There is a decrease in halitosis after the use of photodynamic therapy employing the use of a red dye and blue LED. There is a decrease in halitosis after treatment with probiotics. There is a microbiological change after antimicrobial photodynamic therapy. There is a microbiological change after treatment with probiotics.

*Null hypothesis*: There is no change in halitosis after the use of photodynamic therapy using red dye and blue LED. There is no change in halitosis after treatment with probiotics. There is no microbiological change after antimicrobial photodynamic therapy. There is no microbiological change after treatment with probiotics.

1. **4. OBJECTIVE**

The aim of the present study is to verify whether aPDT treatment, using annatto as a photosensitizer and LED as a light source, is effective in the immediate reduction of halitosis when evaluated by gas chromatography, as well as comparing this method with the use of a tongue scraper, the most commonly used conventional method, dental flossing and teeth brushing with amine fluoride (Elmex®) toothpaste and the use of probiotics.

Perform the quantitative microbiological analysis of the bacteria present in the tongue coating before and after treatment, using real-time PCR.

1. **MATERIALS AND METHOD**

An invitation will be made and participants who are interested in participating will be selected. 52 UNINOVE students or employees diagnosed with halitosis will be included, presenting gas chromatography with sulfide (SH2) ≥ 112 ppb. Participants will be divided by block randomization into four groups (n = 13), according to the treatment to be performed (Figure 1). Group 1: treatment with teeth brushing, dental floss and tongue scraper; Group 2: teeth brushing, dental floss and aPDT applied to the back and middle third of the tongue; Group 3: teeth brushing dental floss and probiotics; Group 4: teeth brushing, dental floss, aPDT and probiotics. The results of halimetry will be compared before, immediately after treatment, seven days after and thirty days after treatment. The microbiological analysis of the tongue coating will be carried out at these same times. Quantitative analysis will be performed using real-time PCR.

This research will be sent to the UNINOVE Research Ethics Committee and because it is a randomized clinical study and seeking greater transparency and quality of this research, we will use the recommendations of CONSORT (Consolidated Standards of Reporting Trials).

1. **1. Inclusion criteria**

Participants in this research will be of both genders, aged 18 to 25 years, diagnosed with halitosis, presenting gas chromatography with sulfide (SH2) ≥ 112 ppb.

- 1. **Exclusion criteria**

Individuals with dentofacial anomalies (such as cleft lip, cleft palates and nasopalatine clefts) will be excluded from the study, those undergoing orthodontic and/or orthopedic treatment, who are undergoing cancer treatment, with systemic changes (gastrointestinal, renal, hepatic), being treated with antibiotics up to 1 month before the survey and pregnant.

**Randomization**

Randomization will be performed using the website randomizer.org, the numbers will be placed in a brown envelope and the participant will remove the envelope at the time of treatment.

- **Procedures**

As this is a randomized clinical study and seeking greater transparency and quality of this research, we will use the CONSORT recommendations (Consolidated Standards of Reporting Trials) (figure 1).

**Participants from UNINOVE dental clinic**

**Excluded**

**Recruitment**

**Halimetry**

**Excluded SH2<112ppb**

**Diagnosis of SH2>112ppb**

**n=52**

**Group 4**

**n=13**

**Group 3**

**n=13**

**Group 2**

**n=13**

**Group 1**

**n=13**

**Teeth brushing, dental floss, aPDT and probiotics**

**Teeth brushing, dental floss and probiotics**

**Teeth brushing, dental floss and tongue scraping**

**Teeth brushing, dental floss and aPDT**

**New halimetry immediately after treatment**

**Halimetry after 7 days**

**Halimetry after 30 days**

**Data analysis**

Figure 1: Activity flowchart.

1. **INTERVENTIONS**

**Group 1 - Brushing, dental floss and scraper**

1- Initial halimetry

2- Collection of tongue coating with swab

3- Tongue scraping

4- Oral Hygiene Guidelines

5- Halimetry immediately after scraping

6- Halimetry after 7 and 30 days

**Group 2 - Brushing, dental floss and aPDT**

1- Initial halimetry

2- Collection of tongue coating with swab

3- Application of annatto spray on the back of the tongue for 2 minutes

4- 6-point LED application

5- Oral Hygiene Guidelines

6- Halimetry immediately after aPDT

7- Halimetry after 7 and 30 days

**Group 3 - Flossing and use of probiotics**

1- Initial halimetry

2- Collection of tongue coating with swab

3- Delivery and guidance on the use of probiotics

4- Oral Hygiene Guidelines

5- Halimetry on the 14th day of treatment with probiotics

6- Halimetry 7 and 30 days after the end of treatment

**Group 4- Brushing, dental floss, aPDT and probiotics**

1- Initial halimetry

2- Collection of tongue coating with swab

3- Application of annatto spray on the back of the tongue for 2 minutes

4- 6-point LED application

5- Delivery and guidance on the use of probiotics

6- Oral Hygiene Guidelines

7- Halimetry on the 14th day of treatment with probiotics

8- Halimetry 7 and 30 days after the end of treatment

**Sample calculation**

To calculate the sample size, data from the paper of Costa da Mota *et al*. (Effect of photodynamic therapy for the treatment of halitosis in adolescents - a controlled, microbiological, clinical trial) was used.

Initially, an error was established, where the baseline groups mean values for periodontal treatment with PDT. From this error, the effect size was calculated, given by

$$\frac{err}{\sqrt{\sigma_{1}^{2}+\sigma_{2}^{2}}}$$

Assuming that the studied groups have a normal or approximately normal distribution, that the sample size will be sufficiently large and that a two-tailed test will be used, for a significance level of α = 0.05 and maintaining the power of the 1-β = 0.90 test. , we have an n = 13 for each group.

Figure 2: Power adjustment in function of total sample size.

Figure 2 shows that with a total sample size of 52 subjects, that is, four groups with 13 samples each, the statistical difference must be demonstrated, maintaining the power of the test greater than or equal to 0.90. If the hypothesis of normality of the distributions is rejected, the sample size should be corrected by approximately 5%.

*Halimetry*

The collection of oral air will follow the manufacturer's instructions (Oral ChromaTM Manual Instruction), where the participant will be instructed to rinse with cysteine (10 mM) for 1 minute, then remain with his/her mouth closed for another minute. A syringe from the same manufacturer to collect oral air will be introduced into the participant's mouth. For 1 minute the participant will remain with his/her mouth closed, breathing through the nose, without touching the syringe with his/her tongue. The plunger will be pulled out, we will again empty the air from the syringe into the participant's mouth and again pull the plunger to fill the syringe with the breath sample. We will clean the tip of the syringe with gauze to remove moisture from the saliva, place the gas injection needle in the syringe, and adjust the plunger to 0.5 ml. The collected gases are injected into the device's entrance door with a single movement (Figure 3).

**Figure 3:** Halimetry process.

From the analysis of the VSCs captured by the system, we have:

- Sulphide: origin mainly of bacteria present on the back of the tongue. Values ​​above 112 ppb are indicators of halitosis.

- Methylmercaptan: predominantly higher in periodontal pockets. Values ​​up to 26 ppb are considered normal. Periodontal disease typically results in a high methylmercaptan/sulfide ratio (> 3: 1).

- Dimethylsulfide: it can be of periodontal origin or of systemic origin (intestinal, hepatic, pulmonary). It can also be caused, temporarily, by eating certain foods and drinks. There is a possibility to distinguish between dimethyl sulfide of oral origin and that of systemic origin, by comparing the results of halimetry with and without the challenge of cysteine ​​(10 mM cysteine, that is, 16 mg of cysteine ​​in 100 ml of water distilled - 16 mg%). The perception threshold for dimethyl sulfide is the lowest, 8 ppb. Other odors (not VSCs) may appear at a peak prior to the theoretically first peak, which is that of the sulfide.

To avoid changes in halimetry, participants will be instructed to follow the following guidelines: 48 hours before the evaluation avoid eating food with garlic, onions and strong spices, alcohol consumption and use of mouthwash. On the day of the evaluation, in the morning, they can eat up to a maximum of 2 hours before the exam, abstain from coffee, candies, chewing gum, oral and personal hygiene products with perfume (aftershave, deodorant, perfume, creams and / or tonic) and brushing will only be done with water.

*Microbiological analysis*

Samples of the tongue coating will be collected using a sterile swab that will be passed on the surface of the back of the tongue with a back and forth motion 10 times. The samples will be deposited in sterile tubes that will be identified and stored at -80 C until analyzed. After thawing, the samples will be vortexed for one minute. For bacterial DNA extraction, the samples will be subjected to a boiling bath for 10 minutes and then centrifuged at 10,000 rpm for 10 minutes. The supernatant will be placed in a new microtube containing 100μL of phenol/chloroform/isoamyl alcohol (25: 24: 1), followed by ethanol precipitation. The purified DNA will be resuspended in TE buffer. The levels of P. gingivalis, T. forsythia and T. denticola will be analyzed by quantitative PCR. Quantitative analysis will be performed using real-time PCR using Step One Plus Real-Time PCR System (Applied Biosystem, Foster City, CA, USA) and products detected by fluorescence using the Quantimix Easy SYG Kit (Biotools, Madrid, Spain), following the protocol recommended by the manufacturer. To the reaction 10 l will be used SYBR Green 0.5 ul DNA template, 200 mM of each primer (P. gingivalis CATAGATATCACGAGGAACTCCGA TT and AAACTGTTAGCAACTACCGATGTGG; T.forsythia GGGTGAGTAACGCGTATGTAACCT and ACCCATCCGCAACCAATAAA, T. denticola CGTTCCTGGGCCTTGTACA and TAGCGACTTCAGGTACCCTCG; Universal bacteria CCATGAAGTCGGAATCGCTAG and GCTTGACGGGCGGTGT) in a total volume of 20 µl. For the standard curve, reactions containing template DNA 2 to 2X105 copies of the analyzed gene (16S rRNA) will be performed using pTOPO plasmids in which the 16S genes of the 14 different organisms will be cloned. As a negative control, sterile milliQ water will be added instead of template DNA. The reactions for 16S rRNA will be carried out with an initial denaturation of 95 ºC for 2 minutes, followed by 36 cycles of 94 ºC for 30 seconds, 55 ºC for 1 minute and 72 ºC for 2 minutes and final extension at 72 ºC for 10 minutes 46. Fluorescence will be detected after each cycle and plotted using Step One Plus Real-Time PCR System software (Applied Biosystem, Foster City, CA, USA). To guarantee the specificity of the products detected by fluorescence and avoid the detection of primer dimers, the detection will be carried out one degree below the dissociation temperature of the amplicons. All samples will be analyzed in duplicate and each dilution of the plasmids to the standard curve in triplicate. The purpose of microbiological evaluation will be to verify the effectiveness of photodynamic therapy for the treatment of halitosis, complementing the clinical evaluation.

*Antimicrobial Photodynamic Therapy (aPDT)*

The LED light curing device - Valo Cordless Ultradent® will be used, an office appliance, with a coupled radiometer, spectrum of 440-480nm and irradiance of 450mW cm. At the time of the aPDT, only the participant to be treated and the responsible professional will be present, both wearing specific glasses for eye protection. The active LED tip will be coated with disposable transparent plastic (PVC) (avoiding cross contamination and for hygiene reasons) and the professional will be properly dressed.

One session of aPDT will be carried out with the annatto photosensitizer (PS) manipulated in 20% concentration (Formula e Ação®) in spray, to be applied in sufficient quantity to cover the middle third and back of the tongue (5 sprays) for 2 minutes for incubation. The excess will be removed with suction in order to keep the surface moist with the PS itself, without using water. 6 points will be irradiated with a distance of 1 cm between the spots, considering the halo of light scattering and effectiveness of aPDT. The device will be previously calibrated with a wavelength 395-480 nm, for 20 seconds per point, energy of 9.6J, and the light will be irradiated so that a halo of 2 cm in diameter per point is formed. Table 1 contains all the parameters used.

Table 1: LED parameters.

| **Wavelength (nm)** | 395-480 |
| --- | --- |
| **Operating mode** | Continuous wave |
| **Average radiant power (mW)** | 480 |
| **Polarization** | random |
| **Aperture diameter (cm)** | 0.9 |
| **Irradiance at aperture (mW/cm2)** | 762 |
| **Beam profile** | Top hat |
| **Beam spot size at target (cm2)** | 3.14 |
| **Irradiance at target (mW/cm2)** | 153 |
| **Exposure duration (s)** | 20 |
| **Radiant exposure (J/cm2)** | 6.37 |
| **Radiant energy (J)** | 9.6 |
| **Number of points irradiated** | 6 |
| **Area irradiated (cm2)** | 18.8 |
| **Application technique** | contact |
| **Number and frequency of treatment sessions** | 1 |
| **Total radiant energy (J)** | 57.6 |

*Tongue scraping*

Tongue scraping will be performed by the same operator in all participants. Posterior-anterior movements will be performed with the scraper over the tongue dorsum, followed by cleaning the scraper with gauze. This procedure will be performed ten times in each participant, in order to standardize the mechanical removal of the tongue coating.

*Toothbrushing with amine fluoride*

All 52 participants will be instructed to brush with toothpaste containing amine fluoride (Elmex®) and dental floss, 3 times a day after meals for 30 days.

*Statistical analysis*

Data from Oral ChormaTM will be analyzed for normality using the Shapiro-Wilk test. If the normality hypothesis is accepted, Analysis of Variance (ANOVA) will be used followed by the Tukey test ,when necessary. To analyze the treatment results in the two study periods, the T test for paired data will be used. If the hypothesis of normality is rejected, the Kruskal-Wallis test will be used followed by the Student-Newman-Keuls test, when necessary. To analyze the results of each treatment in the two study periods, the Wilcoxon test will be used.

1. **DISCUSSION**

Halitosis is considered an important social factor, as it interferes with interpersonal relationships. In addition to raising concerns related to the individual's physical health, it can cause psychological changes, leading to a social barrier [18].

There is a scarcity of studies that assess halitosis reduction through photodynamic therapy and the use of probiotics, presenting annatto as a photosensitizer and LED as a more accessible light source for dentists. Although methylene blue combined with red laser has already been used for this purpose, this study will evaluate the effect of aPDT with annatto and LED to reduce halitosis. The fact that the annatto is red facilitates work combined with a light-emitting diode (LED) and allows its use on a larger scale. In addition, the LED is inexpensive and most dentists already have the device in their offices.

The use of probiotics in dentistry presents an innovative treatment, capable of modifying the oral microbiota, especially with regard to halitosis, since the oral microbiota is complex, being a great challenge in the development of disease prevention and treatment protocols [19].

The present study proposes a controlled clinical trial to compare the effect of photodynamic therapy and the use of probiotics to control halitosis.

**SCHEDULE**

| ***Month/Year →***  ***Activities*** | 03/19 | 04/19 | 05/19 | 06/19 | 07/19 | 08/19 | 09/19 | 10/19 | 11/19 | 12/19 | 01/20 | 02/20 | 03/20 | 04/20 | 05/20 | 06/20 | 07/20 | 08/20 | 09/20 | 10/20 | 11/20 | 12/20 | 01/21 | 02/21 |
| --- | --- | --- | --- | --- | --- | --- | --- | --- | --- | --- | --- | --- | --- | --- | --- | --- | --- | --- | --- | --- | --- | --- | --- | --- |
| ***Ethics Committee Sending*** |  |  |  |  | X |  |  |  |  |  |  |  |  |  |  |  |  |  |  |  |  |  |  |  |
| ***Literature Review*** | X | X | X |  |  |  |  |  |  |  |  |  |  |  |  |  |  |  |  |  |  |  |  |  |
| ***Writing of Materials and Method*** |  |  |  | X | X | X | X | X |  |  |  |  |  |  |  |  |  |  |  |  |  |  |  |  |
| ***Participant recrutiment*** |  |  |  |  |  |  |  |  | X | X | X | X | X | X |  |  |  |  |  |  |  |  |  |  |
| ***Methodology execution*** |  |  |  |  |  |  |  |  |  |  |  |  |  |  | X |  |  |  |  |  |  |  |  |  |
| ***Data analysis*** |  |  |  |  |  |  |  |  |  |  |  |  |  |  |  | X | X |  |  |  |  |  |  |  |
| ***Writing of results*** |  |  |  |  |  |  |  |  |  |  |  |  |  |  |  |  |  | X | X | X |  |  |  |  |
| ***Writing of discussion and conclusions*** |  |  |  |  |  |  |  |  |  |  |  |  |  |  |  |  |  |  |  |  | X | X | X |  |
| ***Send paper for publication*** |  |  |  |  |  |  |  |  |  |  |  |  |  |  |  |  |  |  |  |  |  |  |  | X |

**REFERENCES**

1. ARMSTRONG, Brenda L.; SENSAT, Michelle L.; STOLTENBERG, Jill L. Halitosis: a review of current literature. **American Dental Hygienists' Association**, v. 84, n. 2, p. 65-74, 2010.
2. CALIL, CM.; MARCONDES, FK. Influence of anxiety on the production of oral volatile sulfur compounds. Life Science, v. 79, n. 7, p. 660–4, 10 jul. 2006.
3. SPRINGFIELD, J. et al. Spontaneous fluctuations in the concentrations of oral sulfurcontaining gases. J Dental Res, v. 80, n. 5, p. 1441–1444, 2001.
4. TANGERMAN, A; WINKEL, E. G. The portable gas chromatograph OralchromaTM: a method of choice to detect oral and extra-oral halitosis. J Breath Res, v. 2, n. 1, mar. 2008.
5. TOLENTINO, E. D. S.; CHINELLATO, L. E. M.; TARZIA, O. Saliva and tongue coating pH before and after use of mouthwashes and relationship with parameters of halitosis. J Appl Oral Sci, v. 19, n. 2, p. 90–4, abr. 2011.
6. PORTER, S. R.; SCULLY, C. Oral malodour (halitosis). Bmj, v. 333, n. 7569, p. 632-635, 2006.
7. KARA, C. et al. Effect of Nd: YAG laser irradiation on the treatment of oral malodour associated with chronic periodontitis. Int Dent J, v. 58, p. 151–158, 2008.
8. KARA, C; TEZEL, A; ORBAK, R. Effect of oral hygiene instruction and scaling on oral malodour in a population of Turkish children with gingival inflammation. Int J Paediatr Dent, v. 16, n. 6, p. 399–404, nov. 2006.
9. BICAK, Damla Aksit. A current approach to halitosis and oral malodor-A mini review. The open dentistry journal, v. 12, p. 322, 2018.
10. SUZUKI, Nao et al. Induction and Inhibition of Oral Malodor. Molecular Oral Microbiology, 2019.
11. SCULLY, C.; GREENMAN, J. Halitology (breath odour: aetiopathogenesis and management). Oral diseases, v. 18, n. 4, p. 333-345, 2012.
12. DA CIARCIA, Ana Carolina Costa et al. Action of antimicrobial photodynamic therapy with red leds in microorganisms related to halitose. Medicine, v. 98, n. 1, 2019.
13. KELLESARIAN, Sergio Varela et al. Effect of antimicrobial photodynamic therapy and laser alone as adjunct to mechanical debridement in the management of halitosis: A systematic review. Quintessence International, v. 48, n. 7, 2017.
14. COSTA DA MOTA, Ana Carolina et al. Effect of photodynamic therapy for the treatment of halitosis in adolescents–a controlled, microbiological, clinical trial. Journal of biophotonics, v. 9, n. 11-12, p. 1337-1343, 2016.
15. YOO, Jun-Il et al. The Effect of Probiotics on Halitosis: a Systematic Review and Meta-analysis. Probiotics and antimicrobial proteins, v. 11, n. 1, p. 150-157, 2019.
16. HOPE, Chris K.; WILSON, M. Induction of lethal photosensitization in biofilms using a confocal scanning laser as the excitation source. Journal of Antimicrobial Chemotherapy, v. 57, n. 6, p. 1227-1230, 2006.
17. SALMINEN, Seppo et al. Demonstration of safety of probiotics—a review. International journal of food microbiology, v. 44, n. 1-2, p. 93-106, 1998.
18. ELIAS, Marina Sá; FERRIANI, Maria das Graças Carvalho. Aspectos históricos e sociais da halitose. Revista Latino-Americana de Enfermagem, v. 14, n. 5, 2006.
19. GONÇALVES, Marcela Leticia Leal et al. Photodynamic therapy with Bixa orellana extract and LED for the reduction of halitosis: study protocol for a randomized, microbiological and clinical trial. Trials, v. 19, n. 1, p. 590, 2018.
20. MAHASNEH, Sari; MAHASNEH, Adel. Probiotics: a promising role in dental health. Dentistry journal, v. 5, n. 4, p. 26, 2017.

Informed Consent Form for Participation in Clinical Research:

Name:_____________________________________________________ Adress:___________________________________________________

Phone number:______________City:________________ZIP:________

E-mail:________________________________________________________

**1.Title of Experimental Work:** COMPARATIVE STUDY BETWEEN PHOTODYNAMIC THERAPY WITH URUCUM AND LED AND PROBIOTICS IN THE REDUCTION OF HALITOSIS - RANDOMIZED CONTROLLED CLINICAL TRIAL

**2.Objective:** Assess whether treatment with LED (a light) and a dye (photodynamic therapy), and use of probiotics are effective in treating bad breath.

**3. Justification:** The justification for this research is the search for an alternative to conventional treatment, producing a longer lasting result and more comfort during treatment.

**4. Experimental Phase Procedures:** You are being invited to receive treatment for bad breath, using LED (a light) with a dye to eliminate bacteria or taking probiotics (which are food products that contain live microorganisms whose ingestion benefits), or the conventional method (tongue scraping), at Uninove's clinic.

You will carry out an assessment of the presence or not of bad breath and may participate in one of the four groups of this research according to a draw.

Group 1 will perform tongue scraping and will receive guidance on brushing, dental floss. Group 2 will receive treatment with a dye in spray and blue light, as well as brushing and floss guidance. Group 3 will receive treatment with probiotics and guidance on brushing and flossing. And group 4 will receive treatment with a dye in spray and blue light and also probiotics, in addition to brushing and floss guidance.

Before and after treatment, bad breath assessments and tongue scraping will be performed. Bad breath will be assessed again after 7 days and 3 days.

**5. Discomfort or Expected Risks:** Participants may experience embarrassment about bad breath. Participants who receive dye treatment may experience tongue sensitivity. The probiotic has no risk or side effect because it has microorganisms that are already present in the body.

**6. Protective measures against risks:** The team will be available if the participant shows sensitivity for immediate removal of the product. In order to avoid embarrassment, the evaluation and treatment will be carried out in a reserved room with the presence of only the participant and the researcher.

**7. Research Benefits:** Reception of treatment for bad breath.

**8. Existing Alternative Methods:** None.

**9. Withdrawal of Consent:** the participant is free to withdraw his/her consent at any time and stop participating in the study, without prejudice. It is important to note that in case you give up participating in the study, you will have no prejudice in relation to your academic or professional activities at the university.

**10. Guarantee of Secrecy:** Researchers ensure the privacy of participants regarding the confidential data involved in the research.

**11. Forms of Reimbursement of Expenses resulting from Participation in the Research:** There will be no reimbursement.

**12. Research Location:** The research will be carried out at Uninove Dental Clinic, located at Rua Vergueiro, 235/249 - 2nd subsoil - Liberdade, São Paulo - SP, 01504-001, Telephone: (11) 2633-9000.

**13. Research Ethics Committee** (CEP) is an interdisciplinary and independent collegiate body, which must exist in institutions that carry out research involving human beings in Brazil, created to defend the interests of research participants in their integrity and dignity and to contribute to development of research within ethical standards (Norms and Regulatory Guidelines for Research involving Human Beings - Res. CNS nº 466/12 and Res. CNS 510/2016). The Ethics Committee is responsible for the evaluation and monitoring of research protocols in terms of ethical aspects. Uninove Ethics Committee address: Rua. Vergueiro nº 235/249 - 12th floor - Liberdade - São Paulo - SP CEP. 01504-001 Phone: 3385-9010 comitedeetica@uninove.br. Office hours of the Ethics Committee: Monday to Friday - From 11:30 am to 1:00 pm and from 3:30 pm to 7:00 pm.

**14. Full name and telephone number of Researchers to Contact:** Prof. Dr. Sandra Kalil Bussadori (011) 98381-7453, and student Pamella de Barros Motta- (011) 97397-6458.

**15. Eventual complications that may arise in the course of the research may be discussed by the proper means.**

São Paulo, de de 20__.

**16. Post-Information Consent:**

I, ________________________________________________, after reading and understanding this term of information and consent, I agree that my participation is voluntary, and that I can leave the study at any time, without prejudice. I confirm that I received a copy of this consent form, and authorize the research work and the dissemination of data obtained in this study only in the scientific community.

Participant's Signature

(All pages must be initialed by the research participant)

17. I, ________________________________________ (Responsible researcher), certify that:

a) Considering that research ethics implies respect for human dignity and the protection due to participants in scientific research involving human beings;

 b) This study has scientific merit and the team of professionals duly mentioned in this term is trained, qualified and competent to perform the procedures described in this term;

____________________________________

Signature of the Responsible Researcher
